# A novel necroptosis related gene signature and regulatory network for overall survival prediction in lung adenocarcinoma

**DOI:** 10.1038/s41598-023-41998-2

**Published:** 2023-09-15

**Authors:** Guoyu Wang, Xue Liu, Huaman Liu, Xinyue Zhang, Yumeng Shao, Xinhua Jia

**Affiliations:** 1 Department of Traditional Chinese Medicine, Beijing Shijitan Hospital, Capital Medical University, Beijing, China; 2 Department of Respiration, The Affiliated Hospital of Shandong University of Traditional Chinese Medicine, Jinan, China; 3 Department of General Medicine, The Affiliated Hospital of Shandong University of Traditional Chinese Medicine, Jinan, China; 4 First Clinical Medical College, Shandong University of Traditional Chinese Medicine, Jinan, China; 5 College of Traditional Chinese Medicine, Shandong University of Traditional Chinese Medicine, Jinan, China

**Keywords:** Computational biology and bioinformatics, Gene regulatory networks, Genome informatics

## Abstract

We downloaded the mRNA expression profiles of patients with LUAD and corresponding clinical data from The Cancer Genome Atlas (TCGA) database and used the Least Absolute Shrinkage and Selection Operator Cox regression model to construct a multigene signature in the TCGA cohort, which was validated with patient data from the GEO cohort. Results showed differences in the expression levels of 120 necroptosis-related genes between normal and tumor tissues. An eight-gene signature (CYLD, FADD, H2AX, RBCK1, PPIA, PPID, VDAC1, and VDAC2) was constructed through univariate Cox regression, and patients were divided into two risk groups. The overall survival of patients in the high-risk group was significantly lower than of the patients in the low-risk group in the TCGA and GEO cohorts, indicating that the signature has a good predictive effect. The time-ROC curves revealed that the signature had a reliable predictive role in both the TCGA and GEO cohorts. Enrichment analysis showed that differential genes in the risk subgroups were associated with tumor immunity and antitumor drug sensitivity. We then constructed an mRNA–miRNA–lncRNA regulatory network, which identified lncRNA AL590666. 2/let-7c-5p/PPIA as a regulatory axis for LUAD. Real-time quantitative PCR (RT-qPCR) was used to validate the expression of the 8-gene signature. In conclusion, necroptosis-related genes are important factors for predicting the prognosis of LUAD and potential therapeutic targets.

## Introduction

Lung cancer is one of the cancer types with the highest mortality rates in the world and is the leading cause of cancer-related deaths in men and women^1^. Non-small cell lung cancer (NSCLC) is the most common type of lung cancer, accounting for approximately 85% of all lung cancer cases^2^. Lung adenocarcinoma (LUAD) is an NSCLC subtype and has the highest fatality rate in nonsmokers^3^. Given that LUAD is prone to metastasis and recurrence in the early stage, the prognostic effect of LUAD is extremely poor, and patients with LUAD have an average 5-year survival rate of less than 20%^4^. In clinical practice, the tumor staging system has been widely used in guiding the treatment and prognosis evaluation of cancer patients^5^. However, the prognosis is usually based on inherent anatomical information. Owing to the heterogeneity of LUAD^6^, the development of the disease is difficult to predict. Therefore, effective prognostic biomarkers that can aid clinicians in making accurate LUAD diagnoses, predicting clinical results, and providing references for personalized medicine are urgently needed.

Single genes cannot fully characterize tumors, whereas the overall gene expression pattern of multiple genes can serve as excellent molecular biomarkers. Through machine learning, Tang et al. demonstrated that gene expression data can be used for effective prediction of distant metastasis and survival in nasopharyngeal carcinoma^7^. Survival prediction in LUAD was also related in previous studies, for example, luo et al. established four gene signatures that could predict the prognosis of patients with stage-I LUAD^8^; Cheng et al. established five gene signatures that could predict the prognosis of patients with LUAD, and also found that gliclazide could be used as a potential therapeutic agent for LUAD^9^.

Necroptosis is called programmed necrosis or regulatory necrosis and can be caused by a series of stimuli, ranging from cytokines, viral infections, chemicals, and damage-associated molecular patterns to various forms of physical and chemical cell stress^10^. Through a variety of innate immune signal transduction pathways, cell plasma membrane integrity is lost at an early stage, cell content leaks, and organelles swell. A key feature is the “necrosome” formed by the interaction between a protein kinase, such as RIP1, and RIP3 receptor and through phosphorylation regulated by RIPK3, mixed lineage kinase domain-like (MLKL) is activated, and the activated MLKL transfers from the cytoplasm to the cell membrane and destroys the integrity of the cell membrane, eventually leading to cell necrosis^11^. Caspase-8 inhibits necroptosis by cutting RIPK1, RIPK3, and cylindromatosis (CYLD). Therefore, the occurrence of apoptosis or necroptosis depends on the activity of caspase-8, and blocking it promotes the induction of necroptosis^12,13^. The regulation of cell death by necroptosis has attracted considerable interest in recent years. Necroptosis is an immunogenic cell death pathway that can trigger a strong adaptive immune response and delay tumor progression by enhancing antitumor immune response, but the inflammatory response it triggers may lead to tumor development and metastasis and induce the generation of a tumor immunosuppressive microenvironment^14^. Low necroptosis level leads to tumor formation, and RIPK1, RIPK3, and MLKL expression are significantly reduced in NSCLC pathogenesis^15^.

Necroptosis plays an important role in tumor development and antitumor process, but its specific function in LUAD has not been fully elucidated. Therefore, exploring the relationship between necroptosis and LUAD may develop our understanding of the mechanisms of tumorigenesis and tumor development. Building a prediction signature based on necroptosis-related genes has important clinical significance to the improvement of the survival times of patients with LUAD. Recent studies on necroptosis and lung adenocarcinoma focused on the prognostic signatures of individual mRNAs, miRNAs, or lncRNAs^16–19^, lacking an integrated and comprehensive analysis of ceRNAs and mRNAs for LUAD. In our study, from the perspective of constructing an “mRNA–miRNA–lncRNA” regulatory network, we showed that each RNA has significant prognostic value for LUAD.

The main points of this study design were as follows. Firstly, the differentially expressed genes associated with necroptosis were identified, while the central therapeutic targets as well as their main biological functions were identified with the PPI co-expression network. Secondly, the 8-gene signature model was constructed and patients were divided into risk subgroups for survival analysis, immune infiltration analysis and drug sensitivity prediction. Thirdly, the mRNA–miRNA–lncRNA regulatory network associated with LUAD prognosis was constructed. Finally, we validated the model and the genes by using the GEO database and RT-qPCR. Overall, the necroptosis-related risk signature may be applied to clinical treatment and diagnosis.

## Material and methods

### Information collection

The mRNA expression profile and clinical information of patients with LUAD were downloaded from the TCGA database (https://portal.gdc.cancer.gov/), including the mRNA expression profiles of 54 normal samples and 497 tumor samples. Patients diagnosed with LUAD were excluded if they lacked survival time, survival status, age, and stage. Overall, 486 patients met the screening criteria. RNA-seq data and the clinical information of 440 tumor samples were obtained from the GEO database (https://www.ncbi.nlm.nih.gov/geo/,GSE68465)^20^. The samples were mainly obtained from patients with confirmed LUAD in the United States. The expression data in each database were first normalized to fragments per thousand base million values. Before comparison, the “scale” function in the “limma” R package (version 3.28.14) was used in normalizing data between databases. Meanwhile, we screened and removed the house-keeping genes and low abundance genes. Given that the data from TCGA and GEO are publicly available, this study was exempt from the approval of the local ethics committee. The current research complied with the TCGA and GEO data access policies and publication guidelines.

Necroptosis-related genes were obtained from the KEGG necroptosis signaling pathway (https://www.kegg.jp/pathway/map04217) and the literature^21–25^. After the duplicate values were removed, a total of 161 necroptosis-related genes were retrieved. Specific information is provided in Table S1.

### Identification of differentially expressed necroptosis-related genes

The “limma” R package was used in identifying differentially expressed genes (DEGs) between normal and tumor samples. The false discovery rate was < 0.05, and |Log2(fold change) |> 0 in the TCGA expression data. A heatmap reflecting the expression levels of each DEG between normal and tumor tissues was created using the “heatmap” R package (version 1.0.12). To understand the relationship among the DEGs, we performed a protein–protein interaction (PPI) analysis interaction gene search (STRING) online tool (http://string-db.org/) with a search tool and set the minimum required interaction score to 0.9. Then the result was imported into Cytoscape software (version 3.7.2) for visualization analysis. MCODE cluster analysis was then performed by using “clusterMaker” (degree cutoff = 2, node score cutoff = 0.2, K-core = 2, MAX depth = 100) to identify the most important MCODE clusters according to clustering scores. Core genes were identified using the MCC method in cytoHubba. The results of the two methods were intersected to confirm the core genes. DEGs mutation rates were examined using Cbioportal (http://www.cbioportal.org/).

### Construction and verification of necroptosis-related gene features

We conducted univariate Cox analysis to screen survival-related genes for the development of the risk signature of the least absolute shrinkage and selection operator (LASSO) Cox regression model^26,27^. The “glmnet” (version 4.1) and “survival” (version 3.2) R packages were used^28^. The independent variable in the regression was the standardized expression matrix of the candidate prognosis DEGs, and the response variable was the overall survival (OS) and status of patients in the TCGA cohort. The penalty parameter (λ) of the model was determined through tenfold cross-validation following the minimum standard (that is, the λ value corresponding to the lowest partial likelihood deviation). Ultimately, eight genes were created to model prognosis based on a penalty parameter (λ) determined by the minimum criteria. The risk score was calculated according to the normalized expression level of each gene and its corresponding regression coefficient. The formula as follows: score = e sum (each gene expression × corresponding coefficient). According to the median value of the risk score, the patients were divided into high-risk and low-risk groups. In the comparison of the survival difference between the high-risk and low-risk groups, *P* < 0.05 was the prognostic-related gene. Kaplan–Meier survival curve analysis was used in comparing the OS time between the two groups, and a receiver operating characteristic (ROC) curve was used in assessing the sensitivity and specificity of genetic characteristics. Principal component analysis (PCA) was performed using the “ggplot2” R package (version 3.35) for the verification of classification accuracy. The “Rtsne” package (version 0.15) implements t-Distributed Stochastic Neighbor Embedding (t-SNE) for visual data dimensionality reduction. To verify our model, we obtained data from the GEO database (GSE68465). The risk score for each patient in the GEO cohort was calculated using the formula used in the TCGA cohort. Based on the risk score, patients in the GEO cohort were also divided into low-risk and high-risk subgroups, and the two risk subgroups were compared in terms of OS. To minimize confounding factors, we used propensity score matching (PSM), set the caliper value to 0.3, and calculated the standardized mean difference (SMD) and propensity score values. Univariate and multifactor COX regressions were subsequently conducted to assess whether the risk score could be an independent prognostic factor. We used Gepia2 (http://gepia2.cancer-pku.cn/#index) to observe the relationship between individual gene signatures and OS. The differences in protein levels of eight-gene signature between tumor and normal tissues were analyzed using the Human Protein Atlas (HPA) Website (https://www.proteinatlas.org/).

### Functional enrichment analysis

We carried out a series of gene functional enrichment analyses to determine major biological attributes via the “clusterProfiler” R packages (version 4.2.2), including GO, and KEGG analyses. And we used “quanTIseq” method of the “immunedeconv” R packages (version 2.0.4) to evaluate immune infiltration^29^.

### Significance of the risk signature in antitumor agents

We calculated the IC50 of commonly used chemotherapeutic agents for LUAD in the TCGA database using the “pRRophetic” R package^30,31^, which is a package for predicting clinical chemotherapy response based on oncogene expression levels. We concentrated on antitumor agents that are frequently used for NSCLC treatment, such as cisplatin, paclitaxel, gefitinib, and gemcitabine.

### Prediction of the mRNA–miRNA–lncRNA interactions

We used miRWalk database (http://mirwalk.umm.uni-heidelberg.de/) and Starbase (https://starbase.sysu.edu.cn/index.php) to predict microRNA (miRNA). The results of the two databases were intersected. Then, we used Starbase and DIANA LncBase v3.0 (https://diana.e-ce.uth.gr/lncbasev3) to predict lncRNA^32–34^, and the results of both databases were taken as the intersection. The miRNAs and lncRNAs obtained from the screening should be statistically significant in survival analysis and differential expression analysis of LUAD (*P* < 0.05).

### Cell culture

The LUAD cell lines A549 were purchased from Procell Life Science &Technology Company (CL-0016, Wuhan, China), and the 16HBE cell was purchased from CHI Scientific Inc. (7-1269, Jiangyin, China). 16HBE served as control. 16HBE cells were cultured with RPMI-1640 containing 10% fetal bovine serum in a 37 °C, 5% CO_2_-based incubator, and A549 cells were cultured with Ham’s F-12 K containing 10% fetal bovine serum in a 37 °C, 5% CO_2_-based incubator.

### Real-time quantitative PCR

Total RNA of the cell lines was extracted by the Trizol method, and the concentration of RNA in each sample was determined using a UV spectrophotometer NANO 2000 (Thermo, USA). The cDNA was then transformed by BeyoRT II M-MLV reverse transcriptase (D7160L, Beyotime, Shanghai, China). Based on the SYBR method, gene expression was determined by ExicyclerTM 96 fluorescence quantification (BIONEER, Korea). GAPDH was used as an internal control gene. The expression quantification was obtained with the 2^−△△^Ct method. The primer sequences are shown in Table 1.

**Table 1:** The primer sequences of CYLD, FADD, H2AX, PPIA, PPID, RBCK1, VDAC1, VDAC2 and GAPDH.

Gene	Primer	Sequence	Primer length
CYLD	F primer	TAATAAACCAAAGGCTACAGG	21
	R primer	TGGTGAAGAACGGTCAAAGT	20
FADD	F primer	GAGAAGGCTGGCTCGTCA	18
	R primer	GGAGGTAGATGCGTCTGAGTT	21
H2AX	F primer	TTCCCAGTGGGCCGTGTACA	20
	R primer	CCGCCAGCTCCAGGATCTCA	20
PPIA	F primer	TCCCAAAGACAGCAGAAA	18
	R primer	AGATGCCAGGACCCGTA	17
PPID	F primer	AATCAGAATGGGACAGG	17
	R primer	AACGCACAATTTAGCAG	17
RBCK1	F primer	AAGGACGGCTGCGACTG	17
	R primer	GCAAGGAATCCCATTTACC	19
VDAC1	F primer	TCTTCACCAAGGGCTAT	17
	R primer	GTCGGTATTCCATTTCTC	18
VDAC2	F primer	CTGGGAACAGAAATCGC	17
	R primer	AGCCCTCATAACCAAAGAC	19
GAPDH	F primer	GACCTGACCTGCCGTCTAG	19
	R primer	AGGAGTGGGTGTCGCTGT	18

### Statistical analysis

The Wilcox test was used in comparing the expression levels of genes between normal and tumor samples. The OSs of different groups were compared using Kaplan–Meier analysis with the log-rank test. Univariate and multivariate Cox regression analyses were used in finding independent factors related to survival rate. The unpaired t-test was used to compare the gene expression of the two groups obtained by PCR, the Student’s t-test was used for those that met the chi-square, and the Welch test was used for those that were not chi-square. All statistical analyses were performed using R v4.1.1 and GraphPad Prism 8.2.1.

### Ethics approval

This study was conducted in accordance with the Institutional Animal Care and Use Committee of the Affiliated Hospital of Shandong University of Traditional Chinese Medicine.

## Results

**Figure 1:**
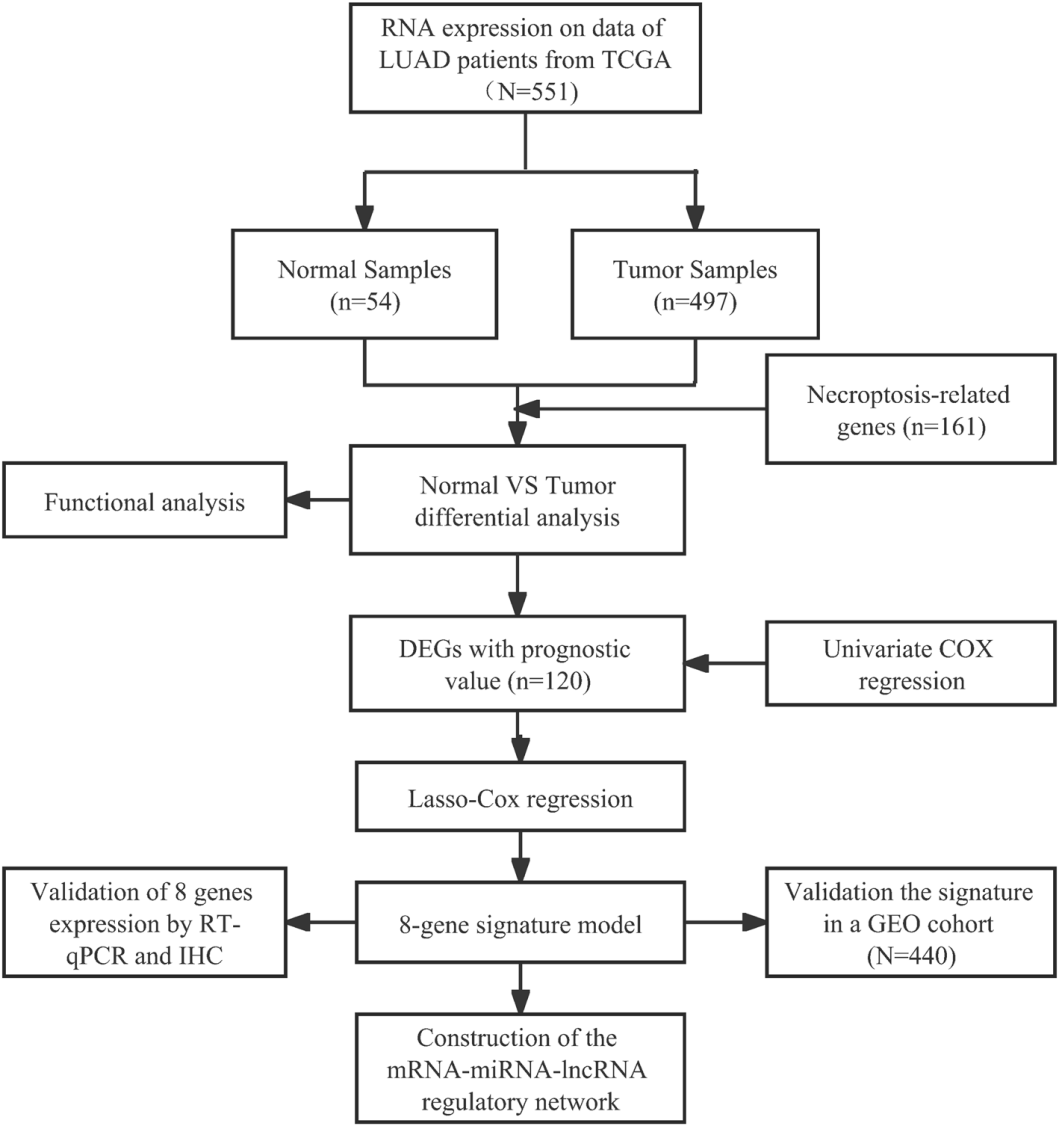
Flow chart of data collection and analysis.

The flow chart of this research is shown in Fig. 1. A total of 486 patients of the TCGA cohort and 440 patients of the GEO cohort were finally included. The detailed information of the patients is shown in Table 2.

**Table 2:** Clinical characteristics of the LUAD patients used in this study.

	TCGA	GEO
Number of patients	486	440
Age (median, range)	66 (33–88)	64 (33–87)
Gender (%)		
Male	264 (54.3)	222 (50.4)
Female	222 (45.7)	218 (49.5)
Stage (%)		
I	262 (53.9)	–
II	112 (23.1)	–
III	79 (16.3)	–
IV	25 (5.1)	–
Unknown	8 (1.6)	–
T (%)		
T1	163 (33.5)	150 (34.1)
T2	260 (53.6)	251 (57.0)
T3	41 (8.4)	28 (6.4)
T4	19 (3.9)	11 (2.5)
Unknown	3 (0.6)	0
M (%)		
M0	333 (68.5)	–
M1	24 (4.9)	–
Unknown	129 (26.6)	–
N (%)		
N0	312 (64.2)	299 (68)
N1	90 (18.5)	87 (19.8)
N2	70 (14.4)	53 (12)
N3	2 (0.4)	0
Unknown	12 (2.5)	1 (0.2)
Survival status		
OS days (median)	653	1577
Alive (%)	304 (62.6)	205 (46.6)
Death (%)	181 (37.4)	235 (53.4)
Unknown	1 (0.2)	0

### Identification of prognostic necroptosis-related DEGs in the TCGA cohort

**Figure 2:**
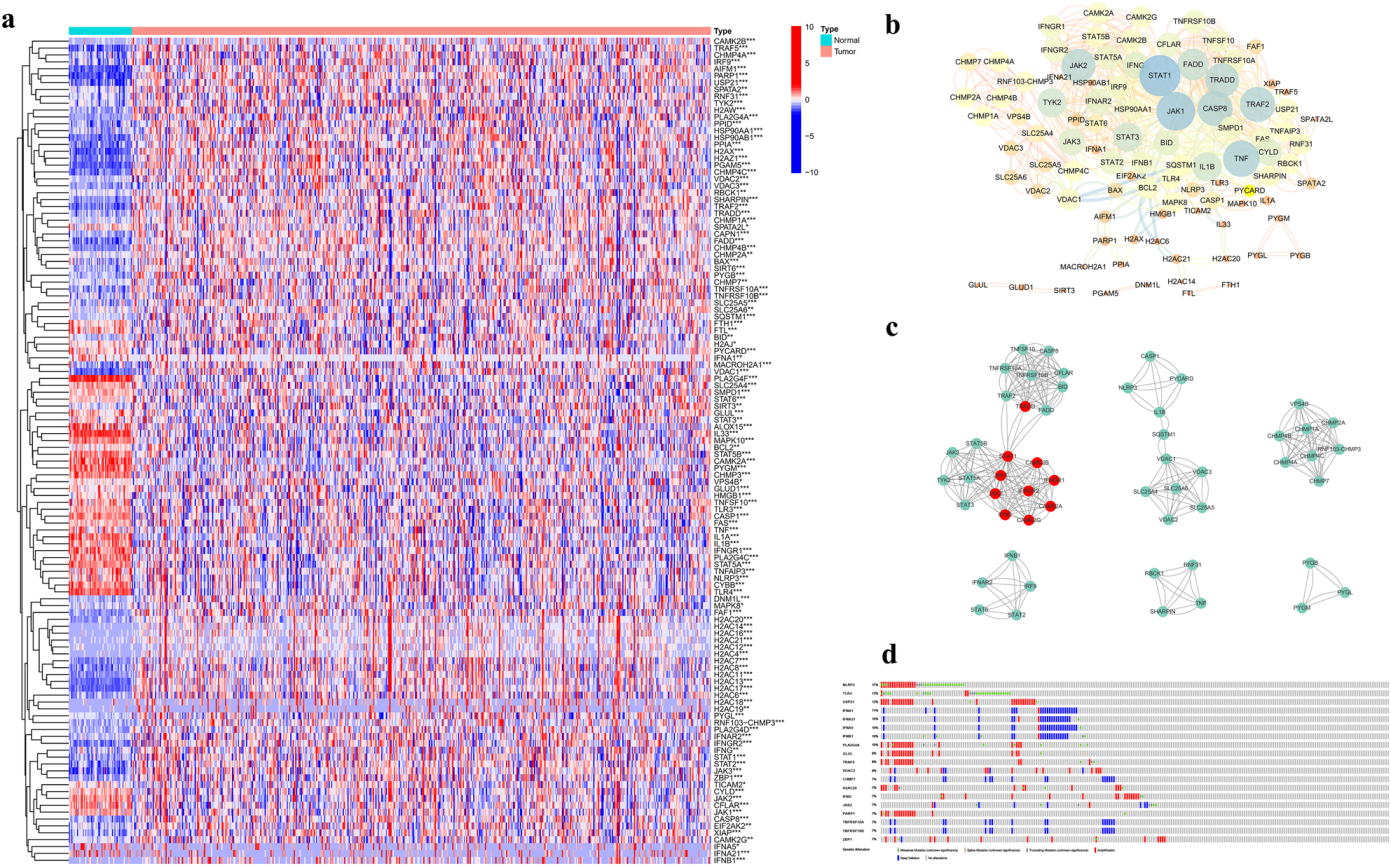
Identification of prognostic necroptosis-related DEGs in the TCGA cohort. (**a**)The heatmaps of 120 DEGs. (**b**) PPI network showing the interactions of DEGs. (**c**) Clustering analysis of DEGs (Top 10 core genes are in red). (**d**) Mutations in DEGs. A total of 19 genes have a mutation rate ≥ 7%.

The expression levels of 54 normal samples and 497 tumor samples in the TCGA database were standardized and corrected, and the expression levels of 161 necroptosis-related genes in the two groups of samples were extracted and compared. Through comparative analysis, we found that 120 genes met the screening criteria. They were differentially expressed necroptosis-related genes (*P* < 0.05). Among them, 77 genes were up-regulated in the tumor samples, and 43 genes were down-regulated in the tumor samples. The results are shown in Fig. 2a. PPI interaction networks were constructed using 120 different necroptosis-related genes (Fig. 2b). The network was divided into 6 clusters with scores of 9.091, 7.714, 4.6, 4, 4, and 3 by MCODE clustering (Fig. 2c). The top ten core genes were identified by cytoHubba, and the clusters with a score of 9.091 were taken to intersect. Finally, we identified JAK1, STAT1, JAK2, IFNGR2, IFNFR1, IFNG, CAMK2G, CAMK2B, CAMK2A, and TRADD as the key genes. We found that missense mutation was the main type of mutation. The top three genes with mutation rates were NLRP3, TLR4, and USP21, which were 17%, 13%, and 13%, respectively. Figure 2d shows the gene with a mutation rate of ≥ 7%.

### Functional enrichment of the DEGs

**Figure 3:**
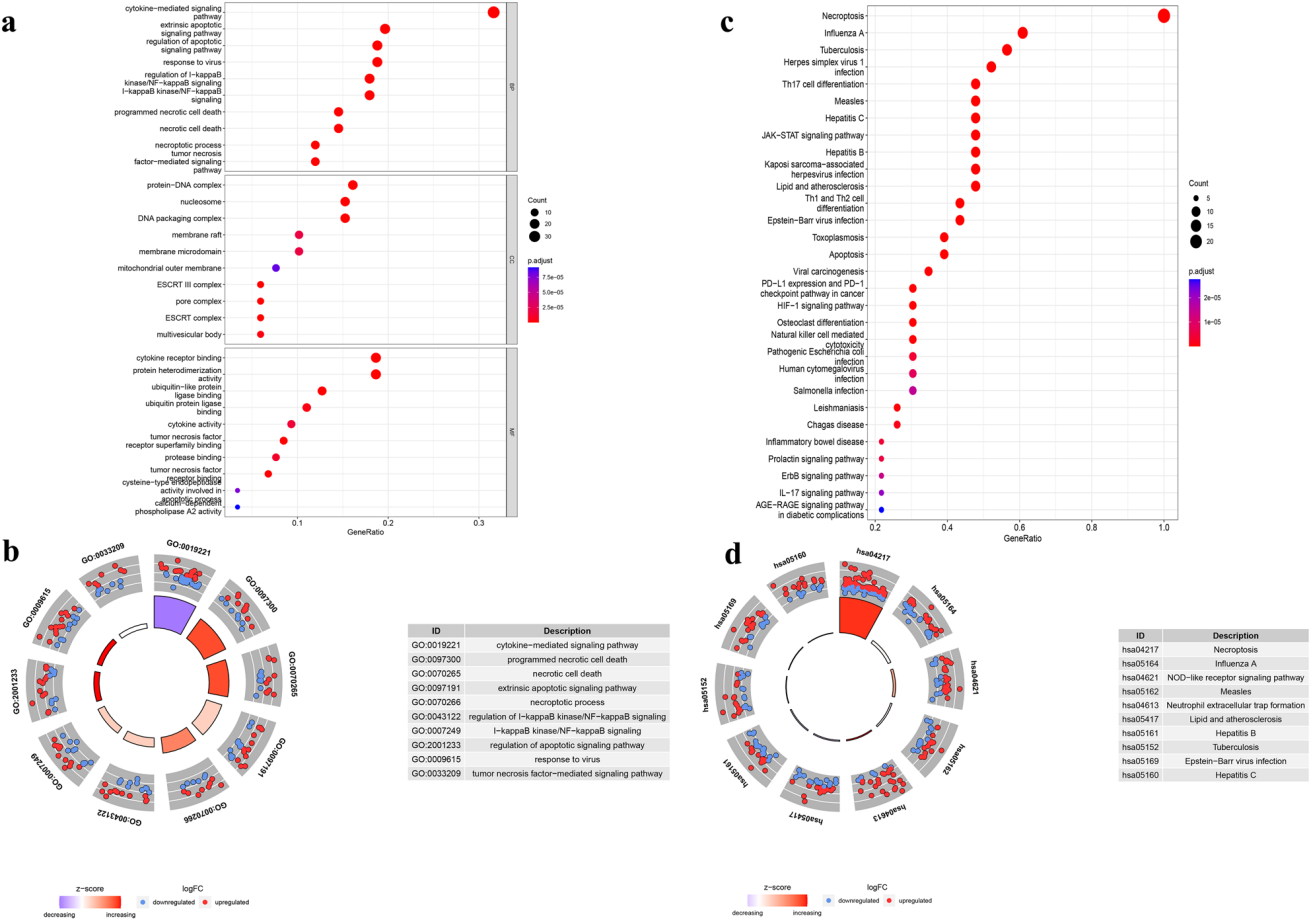
GO and KEGG analysis of DEGs. (**a**) The top 30 significant terms of GO function enrichment. (BP: biological process, CC: cellular component, MF: molecular function). (**b**) The GO circle shows the scatter map of the logFC of the specified gene. (**c**) The top 30 significant terms of KEGG analysis. (**d**) The KEGG circle shows the scatter map of the logFC of the specified gene. The higher the Z-score value indicated, the higher expression of the enriched pathway.

Enrichment analysis was performed for 120 necroptosis-related DEGs. The GO term functional enrichment and the KEGG pathway enrichment analysis of these genes were summarized in Fig. 3. The top enriched GO terms in biological processes were cytokine-mediated signaling pathway, extrinsic apoptotic signaling pathway, regulation of apoptotic signaling pathway, and those in cellular components were the protein-DNA complex and nucleosome. In terms of molecular function, genes were mostly enriched in terms of cytokine receptor binding and protein heterodimerization activity. In the KEGG pathway enrichment analysis, these genes were shown to be associated with pathways related to necroptosis, influenza A, and tuberculosis. Most of the Z-scores of enriched pathways were more than zero, indicating that most of the pathways were likely to be enhanced.

### Construction of a signature-predicting prognosis in the TCGA cohort

**Figure 4:**
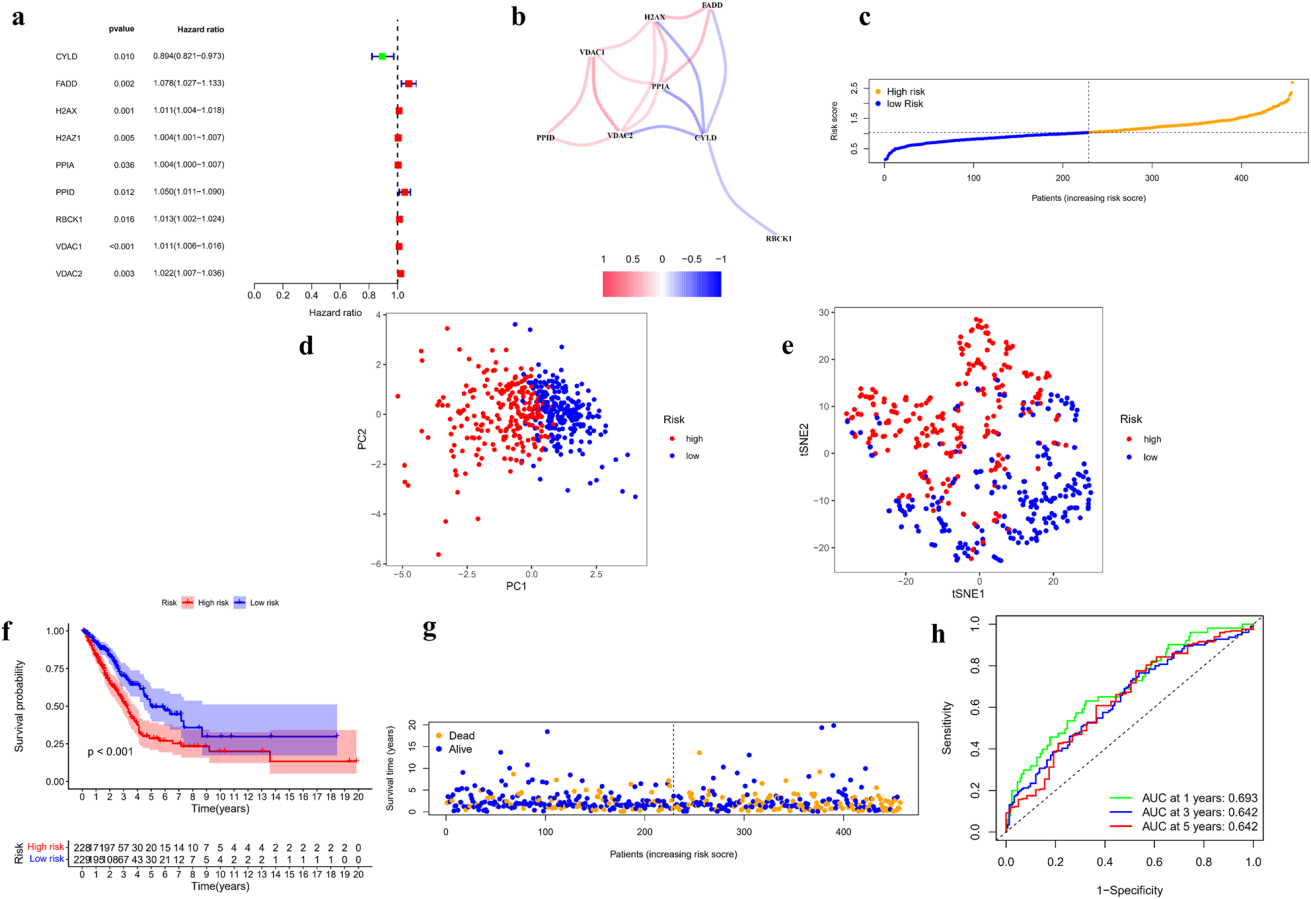
The development of a prognostic index based on DEGs in the TCGA cohort. (**a**) Screen out of the OS-related genes with univariate cox regression analysis. (**b**) The correlation network of the OS-related genes (red is the positive correlation, blue is the negative correlation). (**c**) The risk score for LUADs. (**d**) The PCA plots for LUADs. (**e**) The t-SNE analysis for LUADs. (**f**) Kaplan–Meier curves for the OS of patients in the high-risk and low-risk groups based on the risk score. (**g**) Survival status of LUAD patients. (**h**) The ROC analysis of OS demonstrated the predictive efficiency.

Using the TCGA database, we performed univariate Cox analysis on 120 necroptosis-related DEGs to evaluate the relationship between them and OS and screened nine survival-related genes (*P* < 0.05). The forest diagram of the nine survival genes (CYLD, FADD, H2AX, H2AZ1, PPIA, PPID, RBCK1, VDAC1, and VDAC2) is shown in Fig. 4a. The LASSO regression model was used in establishing a prognostic model for the nine survival genes. According to the penalty parameter (λ) determined by the minimum standard, eight genes, namely, CYLD, FADD, H2AX, PPIA, PPID, RBCK1, VDAC1, and VDAC2, and their coefficients were retained (Fig. S1). The correlation networks of eight gene expression profiles were used in showing the relationships between to the gene expression profiles (Fig. 4b). The risk score was calculated for 457 LUAD patients. According to the median risk scores, 229 patients were divided into low-risk groups, and the remaining 228 patients were high-risk groups (Fig. 4c). The risk score formula was as follows: risk score = (− 0.062*CYLD exp) + (0.044*FADD exp) + (0.005*H2AX exp) + (0.001*PPIA exp) + (0.024*PPID exp) + (0.006*RBCK1 exp) + (0.007*VDAC1 exp) + (0.001*VDAC2 exp). PCA and t-SNE showed that patients with different risks were well separated in two directions (Fig. 4d, e). Through survival difference analysis, we found significant difference in the survival probability between the two risk subgroups (*P* < 0.001, Fig. 4f). We visualized patients in the high-risk group had a higher mortality rate than those in the low-risk group (Fig. 4g). Figure 4h shows the time-dependent ROC curve for the two groups of patients to assess the predictive effect of risk score on OS. The areas under the curve (AUCs) were 0.693 for 1 year, 0.642 for 3 years, and 0.642 for 5 years. In addition, we analyzed eight-gene signature with survival time of patients (Fig. S2). The results showed that, except for CYLD, seven genes were significantly associated with survival time in patients in the high and low risk groups (*P* < 0.05), as well as being risk factors for survival time (HR > 1).

### Independent prognostic value of the eight-gene signature

**Figure 5:**
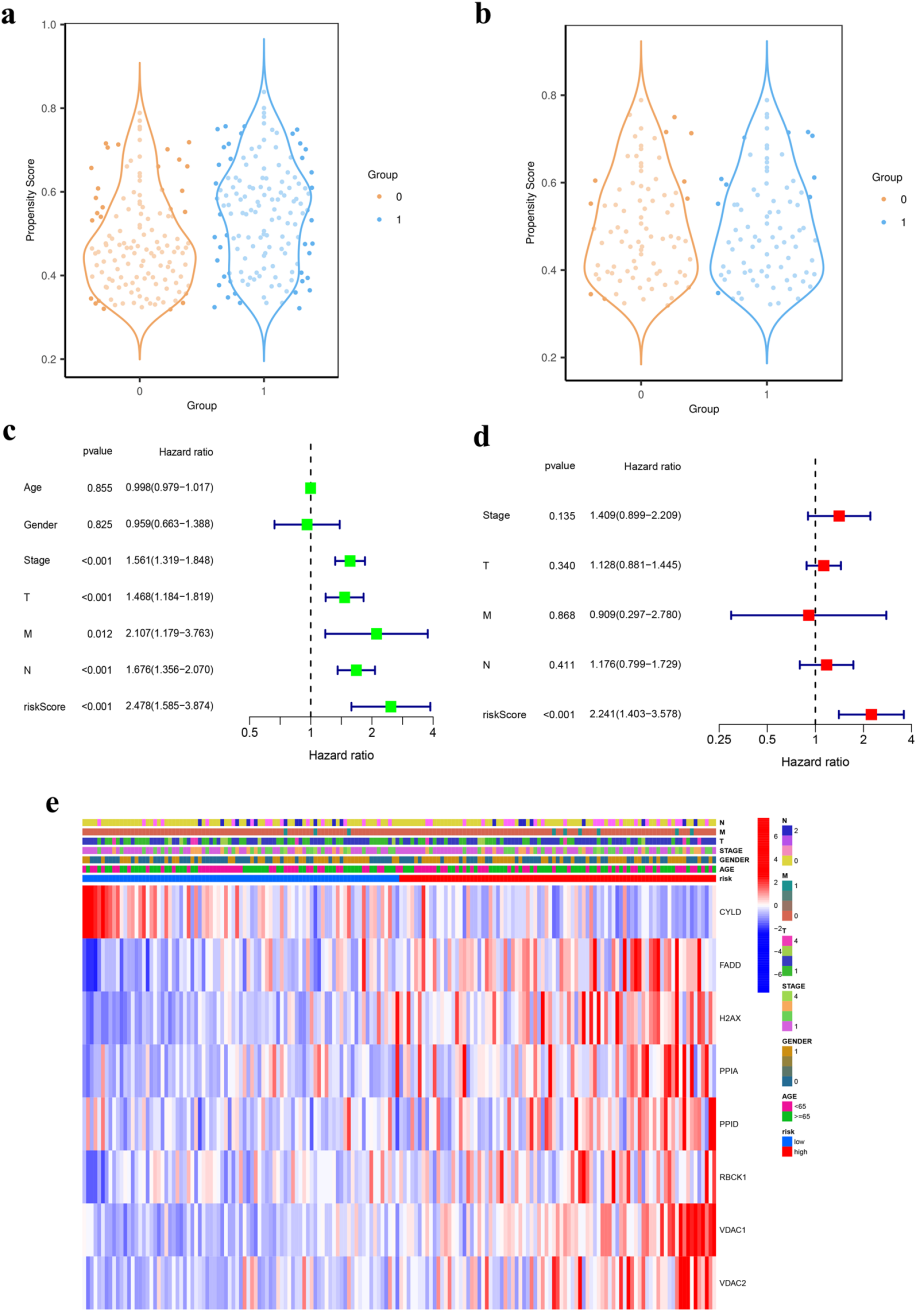
Independent prognostic value of the 8-gene signature. (**a**) Pre-PSM adjusted scores for high- and low-risk groups (0 = low-risk group, 1 = high-risk group). (**b**) PSM adjusted scores for high- and low-risk groups. (**c**) Univariate analysis for TCGA cohort. (**d**) Multivariate analysis for TCGA cohort. (**e**) The heatmap and clinicopathologic features of the 8-gene signature.

To further reduce confounding factors (e.g., radiotherapy, chemotherapy), we used PSM to regroup the sample (Table 3), and the sample propensity scores were shown in Fig. 5a, b. We performed univariate and multivariate Cox regression analyses to determine whether the risk score can be an independent prognostic predictor in the TCGA cohorts. In univariate Cox regression analysis, the risk scores in the TCGA were significantly correlated with OS (HR = 2.478; 95% CI = 1.585–3.874; *P* < 0.001; Fig. 5c). And the risk score was found to be an independent predictor of OS in the multivariate Cox regression analysis (HR = 2.241; 95% CI = 1.403 − 3.578; *P* < 0.001; Fig. 5d). We plotted a heat map to directly show the relationships among the eight genes and the clinical characteristics of the TCGA cohort (Fig. 5e). FADD, H2AX, PPIA, PPID, RBCK1, VDAC1, and VDAC2 were up-regulated in the high-risk group.

**Table 3:** Clinical information and basic characteristics of LUAD patients in TCGA cohort after PSM.

Variables	RISK: low	RISK: high	SMD	*P* value
Age	64.78 ± 10.60	64.16 ± 9.97		0.6988
Gender			0.0707	0.7589
Female	45 (52.9)	42 (49.4)		
Male	40 (47.1)	43 (50.6)		
Treatment				0.6035
No treatment	43 (50.6)	50 (58.8)	0.1660	
Adjuvant chemotherapy	24 (28.2)	17 (20)	0.1934	
Adjuvant radiotherapy	5 (5.9)	6 (7.1)	0.0478	
Adjuvant radio-chemotherapy	13 (15.3)	12 (14.1)	0.0332	
Stage				0.7379
1	47 (55.3)	48 (56.5)	0.0237	
2	23 (27.1)	20 (23.5)	0.0813	
3	12 (14.1)	11 (12.9)	0.0344	
4	3 (3.5)	6 (7.1)	0.1581	
T				0.2701
1	33 (38.8)	24 (28.2)	0.2257	
2	42 (49.4)	54 (63.5)	0.2877	
3	6 (7.1)	3 (3.5)	0.1581	
4	4 (4.7)	4 (4.7)	0.0000	
M			0.1581	0.4933
0	82 (96.5)	79 (92.9)		
1	3 (3.5)	6 (7.1)		
N				0.3920
0	58 (68.2)	54 (63.5)	0.0994	
1	16 (18.8)	23 (27.1)	0.1968	
2	11 (12.9)	8 (9.4)	0.1122

### Validation of the risk signature in the GEO cohort

**Figure 6:**
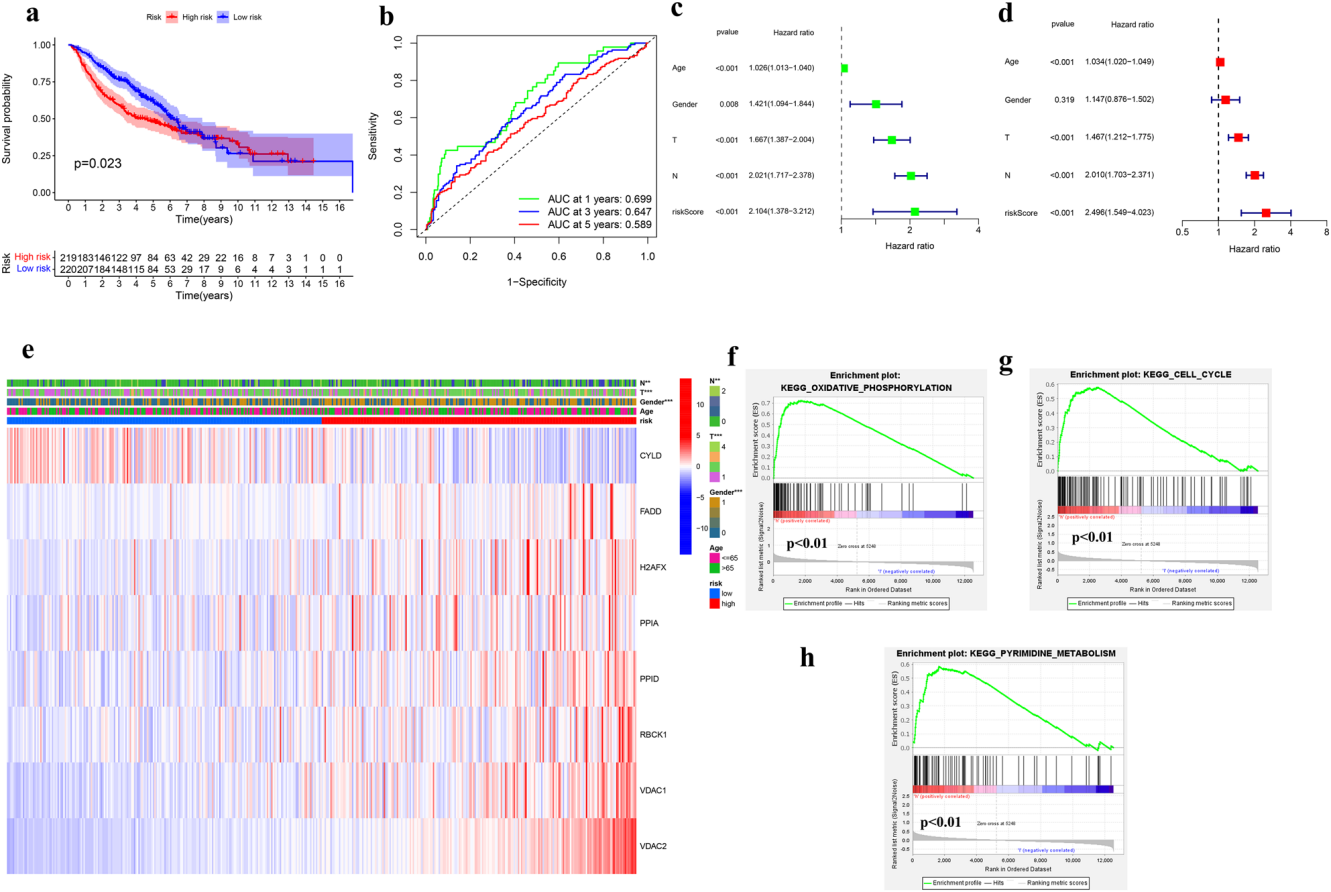
Validation of the risk signature in the GEO Cohort. (**a**) Kaplan–Meier curves for the OS of patients in the high-risk and low-risk groups based on the risk score in the GEO cohort. (**b**) The ROC analysis of OS for the signature in the GEO cohort. (**c**) Univariate analysis for the GEO cohort. (**d**) Multivariate analysis for the GEO cohort. (**e**) The heatmap and clinicopathologic features of the 8-gene signature. (**f**) GSEA validated enhanced activity of Oxidative Phosphorylation. (**g**) GSEA validated enhanced activity of Cell Cycle. (**h**) GSEA validated enhanced activity of Pyrimidine Metabolism.

The complete clinical data of 440 LUAD cases from the GEO database (GSE68465) were included in the external validation set. We normalized the expression data of each gene in the GEO database with the “SCALE” function before verification to prevent deviations caused by different sequencing platforms. Due to the differences in gene annotations across microarrays or sequencing platforms, H2AX was named H2FAX in the GSE68456 dataset, so H2FAX was subsequently used instead. Eight survival-related genes were extracted from the expression profile array. The risk score for each case in the GEO cohort was calculated using the formula used in the TCGA cohort. Using the median risk score in the TCGA cohort as the criteria for risk setting in the GEO cohort, we assigned 220 cases to the low-risk group. The other 220 cases were included in the high-risk group (Fig. S3). PCA and t-SNE analyses showed good separation between the two subgroups. The survival probability of the high-risk subgroup was significantly lower than that of the low-risk group (*P* = 0.023; Fig. 6a). The time-ROC curve revealed the reliable predictive effect of our model in the GEO cohort (Fig. 6b). The AUCs were 0.699 for 1 year, 0.647 for 3 years, and 0.589 for 5 years. As shown in Fig. 6c, after the PSM to exclude confounding factors (Fig. S4, Table S2), the risk score in the GEO cohort was significantly correlated with OS (HR = 2.104; 95% CI = 1.378–3.212; *P* < 0.001) in the univariate Cox regression analysis, and the risk score also was found to be an independent predictor of OS in the multivariate Cox regression analysis (HR = 2.496; 95% CI = 1.549–4.032; *P* < 0.001; Fig. 6d). We plotted a heat map to directly show the relationships among the eight genes and the clinical characteristics of the GEO cohort (Fig. 6e). Differences between the model characteristics of the high-risk and low-risk groups were very significant (Gender and T staging, *P* < 0.001; N staging, *P* < 0.01). FADD, H2FAX, PPIA, PPID, RBCK1, VDAC1, and VDAC2 were up-regulated in the high-risk group. Enrichment analysis of GEO cohort data with GSEA (Fig. 6f–h) revealed that necroptosis-related pathways are closely related to oxidative phosphorylation, cell cycle, and pyrimidine metabolism.

### TCGA and GEO immune infiltration

**Figure 7:**
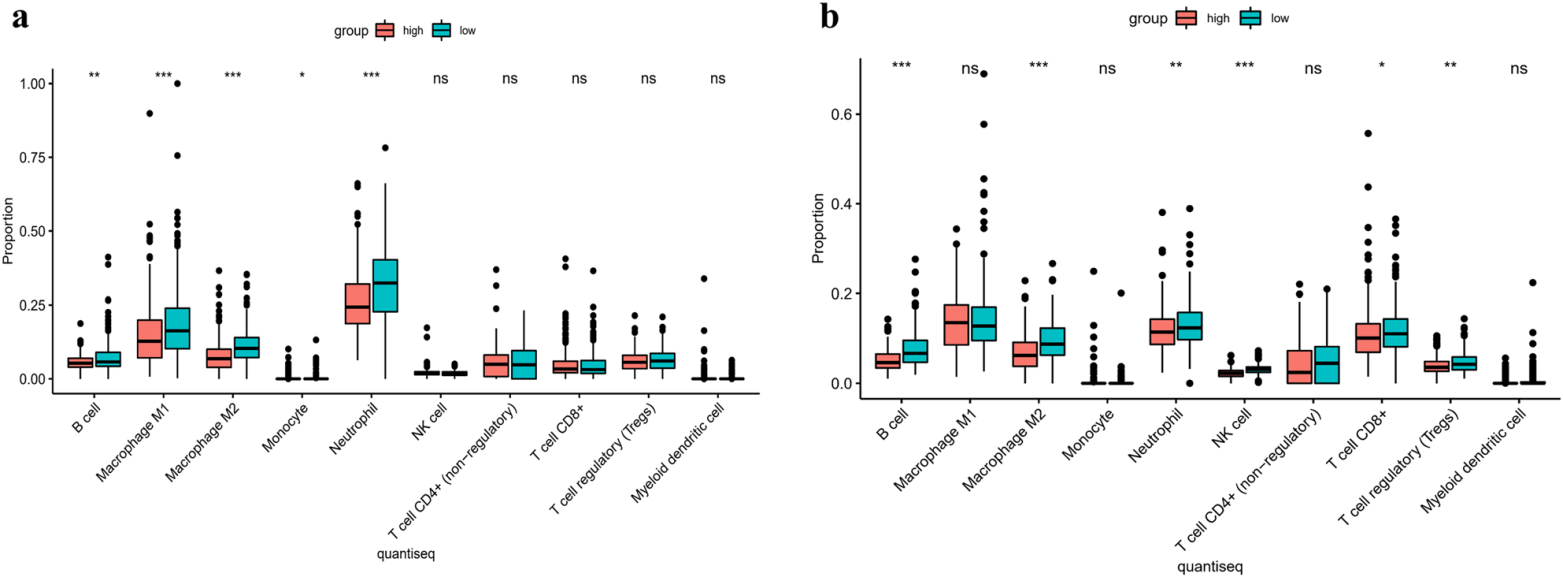
The proportion of 10 cell types of DEGs in the risk subgroups in the TCGA cohort (**a**) and GEO cohort (**b**). (*P* values were showed as: *, *P* < 0.05; **, *P* < 0.01; ***, *P* < 0.001).

In the TCGA cohort (Fig. 7a), the expression levels of immune cells, such as B cell, macrophages M1, macrophages M2, monocyte, and neutrophils significantly varied between the high-risk and low-risk groups (*P* < 0.05 or *P* < 0.001). The proportion of immune infiltration of the high-risk group was lower than that of the risk group. In the GEO cohort (Fig. 7b), the proportion of Macrophages M2, neutrophils were lower in the high-risk group, especially in antigenic cells, such as B cell, T cell CD8+, and Treg (*P* < 0.05). These results suggested that necroptosis-related genes are correlated with the immune status of patients with LUAD.

### Association of the risk signature with antitumor agents

**Figure 8:**
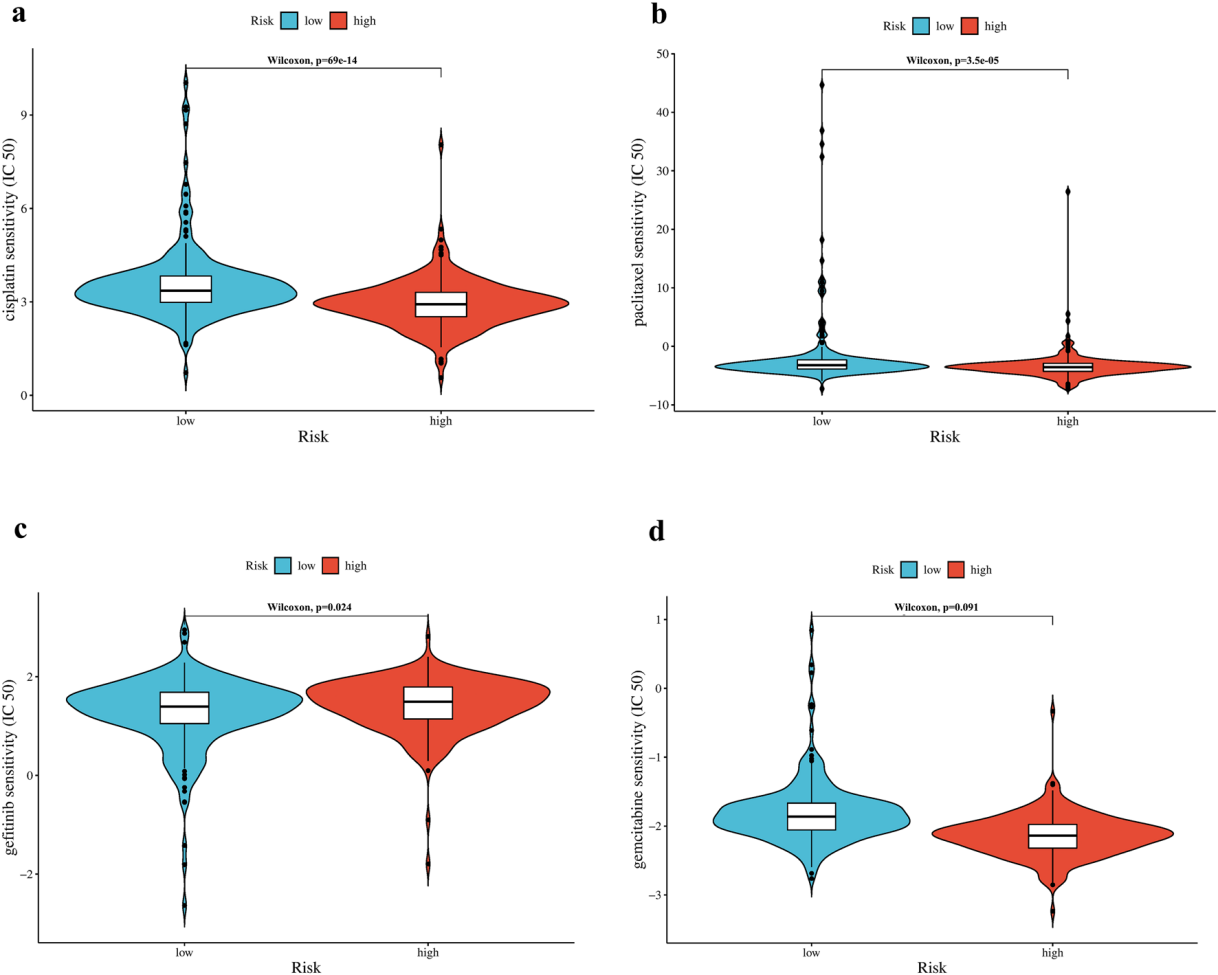
Prediction of sensitivity of antitumor agents. IC50 of drugs was calculated by gene expression levels of patients and drug sensitivity data in cancer cell lines. IC50 was calculated for cisplatin (**a**), paclitaxel (**b**), gefitinib (**c**), and gemcitabine (**d**) in high-and low-risk group.

We applied the “pRRophetic” R package to analyze gene expression sequencing data, focusing on cisplatin, paclitaxel, gefitinib, and gemcitabine, which are frequently used for NSCLC treatment. As shown by the half-inhibitory concentration (IC50), a significant difference was observed in sensitivity to paclitaxel, cisplatin, and gefitinib between the high- and low-risk groups (Fig. 8, *P* < 0.05), while the difference was not significant in gemcitabine. These results suggested that our risk signature might guide the use of antineoplastic agents in the clinical setting.

### Construction of the mRNA–miRNA–lncRNA regulatory network

**Figure 9:**
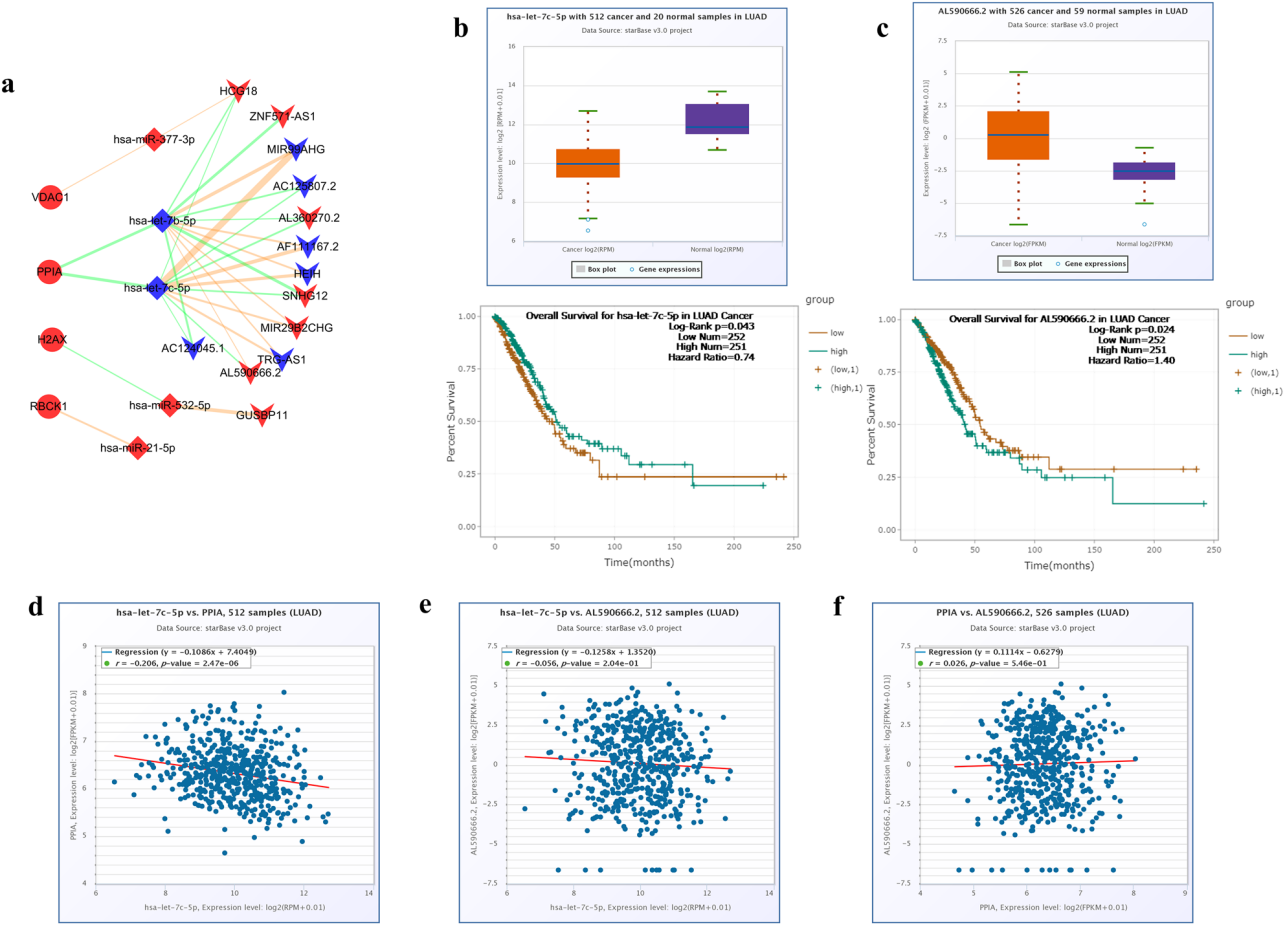
Construction of the mRNA–miRNA–lncRNA regulatory network. (**a**) The mRNA–miRNA–lncRNA regulatory network (The circle represents mRNA, the diamond represents miRNA, the V-shaped arrow represents lncRNA, the red represents high expression in LUAD, the blue represents low expression in LUAD, the orange represents positive correlation, and green represents negative correlation, *P* < 0.05). (**b**) The differential expression and survival analysis of let-7c-5p in LUAD. (**c**) The differential expression and survival analysis of lncRNA AL590666.2 in LUAD. (**d**) Expression correlation between let-7c-5p and PPIA. (**e**) Expression correlation between let-7c-5p and lncRNA AL590666.2. (**f**) Expression correlation between lncRNA AL590666. 2 and PPIA.

We obtained 155 miRNAs from the miRWalk and Starbase, and validated them in the Starbase database for differential gene expression and survival analysis in LUAD. We finally obtained 5 miRNAs (hsa-let-7c-5p, hsa-let-7b-5p, hsa-miR-21-5p, hsa-miR-377-3p, and hsa-miR-532-5p, Fig. S5, *P* < 0.05). All of them, hsa-let-7c-5p and hsa-let-7b-5p were lowly expressed, and hsa-miR-21-5p, hsa-miR-377-3p, and hsa-miR-532-5p were highly expressed. Then we obtained 13 lncRNA (MIR29B2CHG, SNHG12, AC124045.1, AC125807.2, AF111167.2, AL360270.2, AL590666.2, HCG18, HEIH, MIR99AHG, ZNF571-AS1, GUSBP11, and TRG-AS1) based on the Starbase database and DIANA LncBase v3.0 (Fig. S6, *P* < 0.05). According to the hypothesis of ceRNA, the correlation of gene and miRNA expression, as well as of lncRNA and miRNA, should be negative^35^. We finally obtained a regulatory network consisting of 4 mRNA (H2AX, RBCK1, PPIA, VDAC1), 5 miRNA and 13 lncRNA (Fig. 9a). As shown in Fig. 9b, c, we found that the let-7c-5p is low expressed and AL590666.2 is high expression in LUAD. Meanwhile, let-7c-5p showed a negative expression correlation with PPIA and AL590666.2, and PPIA showed a positive expression correlation with AL590666.2 (Fig. 9d–f). Hence, we combined the correlation coefficients to speculate that lncRNA AL590666.2/let-7c-5p/PPIA is a regulatory axis, which has important roles in the development of lung adenocarcinoma. The lncRNA AL590666.2 could competitively bind let-7c-5p to affect the expression of PPIA. However, further in vivo and in vitro experiments are still needed to verify.

### Real‑time quantitative PCR

**Figure 10:**
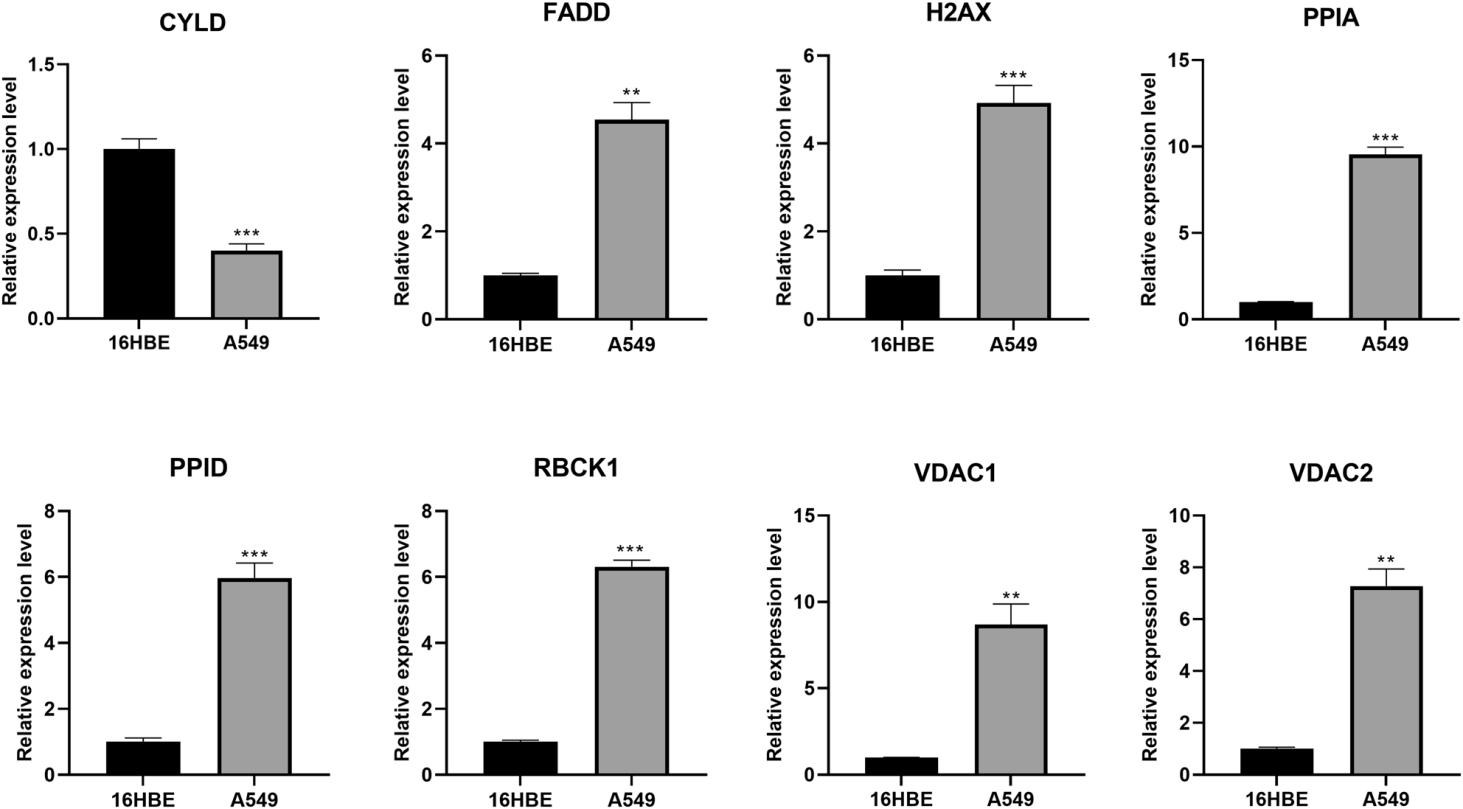
RT-qPCR validate the expression of the 8-gene signature. (*P* values were showed as: *, *P* < 0.05; **, *P* < 0.01; ***, *P* < 0.001).

To further validate the expression of the 8-gene signature in the model, we examined the expression of genes in normal and LUAD cells by rt-qPCR. The results were consistent with our results in the TCGA cohort data and GEO cohort data (Fig. 10), CYLD was significantly less expressed in A549 cells than in 16HBE cells (*P* < 0.001), whereas the expression of the other 7 genes (FADD, H2AX, PPIA, PPID, RBCK1, VDAC1, VDAC2) was significantly higher in A549 cells than in 16HBE cells (*P* < 0.001 or *P* < 0.01). Immunohistochemical (IHC) data from the HPA database showed that CYLD protein levels were also significantly down-regulated in LUAD tissues (Fig. S7), whereas the protein levels of FADD, H2AX, PPIA, PPID, VDAC1, and VDAC2 were significantly up-regulated in lung tissues.

## Discussion

In this study, the differential analysis of 161 necroptosis-related genes in the TCGA database revealed that 120 DEGs have differential expression levels in normal and tumor tissues. OS and LASSO regression models were used, and univariate Cox regression analysis was performed on 120 DEGs for the screening of eight prognostic-related genes, and a risk model was established. The model was a strong predictor of patient prognosis in the TCGA and GEO cohorts, and functional analysis indicated the enrichment of immune-related pathways. Low immune activation was found in high-risk LUAD cases.

The prognostic model in this study consisted of eight necroptosis-related genes (RBCK1, CYLD, FADD, H2AX, PPIA, PPID, VDAC1, and VDAC2). In the TNFα-induced necroptosis, TNF receptor-1 (TNFR1) binds to TNFα, whereas TNFR1 homodimerization produces the TNFR1-associated DEATH domain protein, which forms a complex with TNFR2, RIPK1, and cellular inhibitors of apoptosis proteins 1 and 2 (cIAP1/2) and activates the NF-κB pathway^36^.

RBCK1 is one of the RING-in-Ring E3 ubiquitin ligases, which can ubiquitinate RIPK1 in conjunction with cIAP1/2 and activate the NF-κB pathway for cell survival. RBCK1 exerts carcinogenesis effects^37^, and the expression level of RBCK1 is regulated by HIF. Experimental evidence shows that the silent expression of RBCK1 impairs the growth of lung tumors^38^.

CYLD, a tumor-suppressor gene, can mediate cell death by regulating the expression of the NF-κB prosurvival gene or the ubiquitination status of RIPK1^39^. It is under-expressed in pancreatic cancer, breast cancer, colon cancer, and other tumors^40–42^. This study found that CYLD is under-expressed in LUAD and has a negative regulatory effect on NF-κB. The initiator of inflammation-associated tumors^43–45^ and down-regulation of CYLD may promote tumor metastasis.

FADD is a death domain protein that binds specifically to the Fas cytoplasmic region. During necroptosis activated by TNF-α, RIPK1 fails to ubiquitinate because of the inhibition of cIAP1/2 by the second mitochondria-derived activator of caspases, resulting in the release of complex 1 and the recruitment of Fas and FADD to form complex II. Complex II activates caspase-8 activation and promotes apoptosis^36^. RIPK1 acts as a scaffolding protein that inhibits apoptosis in FADD-caspase8-dependent cells and RIPK3-MLKL-dependent cells and promotes cell survival^46^.

H2A is a histone protein present in chromosome nucleosomes that bind to DNA, and H2AX (H2AFX) is a variant of H2A^47^. DNA damage leads to the phosphorylation of H2AX and ultimately results in the lysis of chromatin. DNA damage caused by environmental factors is one of the main triggers for the transformation of normal cells into tumor cells, and DNA damage is significantly increased in tumor tissues compared with that in normal tissues. γ-H2AX can be used as a prognostic indicator in predicting the prognosis of NSCLC. Immunohistochemistry findings of high γ-H2AX expression levels indicate aggressive and highly proliferative tumors with poor prognoses^48–50^. This result is consistent with the results of the present study, in which patients with LUAD in the high-risk group showed high expression of H2AX.

PPIA belongs to a family of immunoaffinity proteins and its secretion is associated with hypoxia, infection, and oxidative stress and has an important role in protein folding, transport, and T-cell activation^51^. PPIA up-regulation is a key factor in cancer transformation and metastasis and is regulated by p53 and HIF1α^52–54^. Another report showed that PPIA could promote cancer metastasis in NSCLC through p38 MAPK^55^. In addition, the expression of PPIA is affected by chemotherapeutic drugs, and the overexpression of PPIA can render an organism resistant to chemotherapeutic drugs, which in turn become ineffective^56,57^.

PPID, VDAC1, and VDAC2 are mitochondrial regulators, and PPID is one of the key proteins for cell death^58^. Elevated PPID phosphorylation causes mitochondrial permeability to excessive pore openings, leading to the development of necroptosis^59^. The up-regulation of PPID protein induced by oncogenic Ras through the Raf-1/MEK/ERK pathway has a decisive role in tumor progression^60^. VDAC1 mediates the regulation of apoptosis by mitochondrial apoptosis proteins, and elevated VDAC1 promotes the development of NLCLC^61^. This finding is consistent with the results of the present study, in which patients in the high-risk group had significantly higher VDAC1 expression and higher mortality. The interaction between Mcl-1 and VDAC promotes lung cancer cell migration^62^.

Gene mutation results showed that NLRP3, TLR4, and USP21 were the top three genes with mutation rates. NLRP3, as an inflammatory vesicle, is the most characteristic of the inflammatory vesicle family. Necroptosis is closely related to the inflammatory response, and NLRP3 leads to necroptosis generation^63^. NLRP3 has dual pro-tumorigenic and protective anti-tumorigenic effects in different types of tumors^64^. The report has shown that NLRP3 has a pro-tumorigenic role in lung cancer^65^. TLR4 has a vital role in both inflammation and the immune system, and its activation triggers the production of pro-inflammatory cytokines. Chronic inflammatory stimuli have a promoting effect on tumors. Studies have found that TLR4 expression was positively correlated with tumor differentiation in lung cancer patients, and patients with TLR4 overexpression had a poorer prognosis^66,67^. USP21 can promote tumorigenesis by increasing the cell proliferation, migration, and invasion of non-small cell carcinoma cells^68^.

The NOD-like receptor signaling pathway is the most potential underlying biological pathway involved in necroptosis and LUAD progression in our research. NOD2 is a member of the NLR family and plays an important role in both innate and adaptive immune responses, apoptosis, autophagy and reactive oxygen species generation. After recognizing the corresponding pattern molecules, NLR can form large signaling molecules through self-oligomerization, such as NLRP3, NLRC4 and other inflammatory vesicles, NOD2 and other Nodosome, which activate the NF-κB pathway, MAPK pathway, cellular death, and release inflammatory cytokines, including TNFα, IL-1β and IL-18, which mediate a series of downstream immune-inflammatory cascade responses^69,70^. Recent studies have found that NOD2 may be involved in the development and treatment of cancer^71^. In addition, necroptosis is downstream of the tumor necrosis factor (TNF) receptor family and also interacts with inflammatory vesicle activation induced by the NOD-like receptor pyrin 3 (NLRP3)^72^.

The GESA enrichment result of GEO showed that oxidative phosphorylation, cell cycle, and pyrimidine metabolism The cell cycle plays an important role in cell death. Enhanced cell cycle in cancer cells suppresses antitumor immunity^73^. The report found that arresting the cell cycle in mitosis can promote the generation of necroptosis^74^. CYLD negatively regulates the cell cycle by controlling cell growth and division at the G(1)/S-phase and cytokinesis by associating with alpha-tubulin and microtubules through its CAP-Gly domains^75^. Increased expression of phosphorylated FADD causes cell cycle dysregulation, which may explain the low survival rate of lung adenocarcinoma patients^76^. The study found that PPIA can promote the transition from G1 to S phase in the liver cancer cell cycle, which may be the reason why PPIA can promote tumor growth^77^. Research has found that PPID accelerates cell cycle progression, promotes cell proliferation, and leads to cell migration and invasion^78^. In addition, we found more metabolic changes in the GEO enrichment results. Tumor cells undergo metabolic changes that are different from those of normal cells, and they can adapt to the altered metabolic environment by switching between glycolysis and oxidative phosphorylation. Oxidative phosphorylation is carried out in mitochondria, a key regulator of necroptosis that plays a pro-inflammatory and immune response-enhancing role^79^. Pyrimidine metabolism has a promotional role in cancer cell proliferation, and mutant p53 can regulate gene expression in pyrimidine metabolism^80^.

In the present study, a significant difference in the content of antigen-presentation process between the low-risk and high-risk groups was found, suggesting that necroptosis promotes the activation of tumor immunity. The finding is consistent with previous studies^81^. However, the low expression of immune cells in high-risk patients compared with patients in the low-risk group may be related to the tumor immune escape mechanism. Tumor cells can reduce antigen-presentation and evade immune recognition, disrupting antigen processing and presenting down-regulated tumor cell expression of HLA-1 and promoting tumor development or even metastasis^82^. It is known that macrophage M1 has a pro-inflammatory effect and macrophage M2 has an anti-inflammatory effect. The low expression of macrophage M2 in patients in the high-risk group indicates that the anti-inflammatory capacity of the body is diminished. Macrophage M2 was found to have an inhibitory effect on necroptosis^83^. Jackute^84^ found that high M2 macrophage infiltration in tumors is associated with reduced overall survival in NSCLC. In addition, massive M2 macrophage infiltration leads to a poor survival prognosis in tumor diseases such as uveal melanoma, pancreatic cancer, and gastric cancer^85–87^. High-density tumor-infiltrating Tregs may promote hepatocellular carcinoma progression and reduce survival by promoting angiogenesis^88^. In addition, Tregs were significantly elevated in the tissues of gastric cancer patients, affecting the survival prognosis of gastric cancer patients^89^. The pRRophetic analysis showed that the high-risk group was sensitive to cytotoxic chemotherapy such as cisplatin and paclitaxel, in contrast to the low-risk group, which was sensitive to gefitinib. It was also consistent with previous studies that cisplatin and paclitaxel showed better drug sensitivity in the high-risk group^90,91^, confirming the accuracy of our study. However, considering the prognosis, the data seem contradictory. Macrophage M2-related gene drug sensitivity studies found that the high-risk group responded better to paclitaxel and paclitaxel^92^. We considered that this is closely related to the different levels of immune infiltration in the high- and low-risk groups. It was found that an increased proportion of M2 TAM promoted the development of tumor immune escape and chemoresistance^71^. Gefitinib had better sensitivity for patients in the low-risk group, and we compared EGFR expression in the high- and low-risk groups and found that it was higher in the low-risk group relative to the high-risk group.

And we identified lncRNA AL590666. 2/let-7c-5p/PPIA as a regulatory axis, which may play a vital role in the progression of LUAD. Overexpression of let-7c-5p increased proliferation, migration, and invasion of LUAD and promoted the production of apoptosis^93,94^. A negative correlation between let-7c-5p on pro-inflammatory factors such as IL-1β and TNF-α was found^95^. Meanwhile, the anti-inflammatory effect could be enhanced by increasing the expression of let-7c-5p. However, the relationship between lncRNA AL590666. 2 and lung adenocarcinoma has not been reported yet.

To verify the accuracy and credibility of our prognostic model genes, we examined the expression of these eight genes in the 16HBE cell line and A549 cell line, respectively. As in previous studies, CYLD expression was significantly lower in A549 cells than in 16HBE cells, while FADD, H2AX, PPIA, PPID, RBCK1, VDAC1, VDAC2 expression was significantly higher in A549 cells than in 16HBE cells. Hence, we concluded that necroptosis is dysfunctional in the high-risk population and that the impaired immune function associated with high-risk patients with LUAD is an important cause of poor prognosis. Therefore, exploring the specific mechanisms between necroptosis and immunity improves the survival of patients with LUAD.

Constructing a necroptosis apoptosis-related gene risk model in this study can help clinics effectively predict the overall survival of patients and conduct a risk assessment, which can help guide the use of clinical antitumor drugs. In addition, this study constructed a necroptosis-associated apoptosis regulatory network, which has diagnostic and prognostic value, could be a potential diagnostic biomarker, and potentially play an important role in the pathogenesis and development of LUAD.

However, our study has several limitations and shortcomings. First, it would be desirable to include more clinical databases for external validation. Second, the potential molecular mechanisms and functions of the regulatory network of LUAD should be further verified by experiments. Therefore, we will collect and expand clinical samples in subsequent work and try to validate the accuracy of the model through more external experiments.

## Conclusion

Our study showed that the expression of most necroptosis-related genes was differentially expressed in normal and tumor samples, suggesting that necroptosis is closely associated with the survival of patients with LUAD and the progression of LUAD. Meanwhile, we constructed and validated an eight-gene signature associated with necroptosis that was shown to be independently associated with OS and accurately predicted the prognosis of patients with LUAD. In addition, necroptosis-related genes in LUAD were correlated with tumor immunity, but reports on their detailed mechanisms are few. Further studies are still necessary. And we constructed a mRNA–miRNA–lncRNA regulatory network, which identified lncRNA AL590666. 2/let-7c-5p/PPIA as regulatory axis for LUAD. In conclusion, we established a novel prognostic model of eight necroptosis-related genes, which provide novel markers for assessing the prognosis of LUAD and provide important evidence for future studies on the mechanisms in necroptosis-related genes and immunity to LUAD.

### Supplementary Information

Supplementary Figure S1.

Supplementary Figure S2.

Supplementary Figure S3.

Supplementary Figure S4.

Supplementary Figure S5.

Supplementary Figure S6.

Supplementary Figure S7.

Supplementary Table S1.

Supplementary Table S2.

## Data Availability

Publicly available datasets were analyzed in this study. This data can be found at TCGA project (https://portal.gdc.cancer.gov/), and the GEO database (https://www.ncbi.nlm.nih.gov/geo/,GSE68465).
