# Supplementary figures and images for: A novel necroptosis related gene signature and regulatory network for overall survival prediction in lung adenocarcinoma
Source: Sci Rep. 2023 Sep 15;13:15345. doi: 10.1038/s41598-023-41998-2 (PMC10504370; doi:10.1038/s41598-023-41998-2)

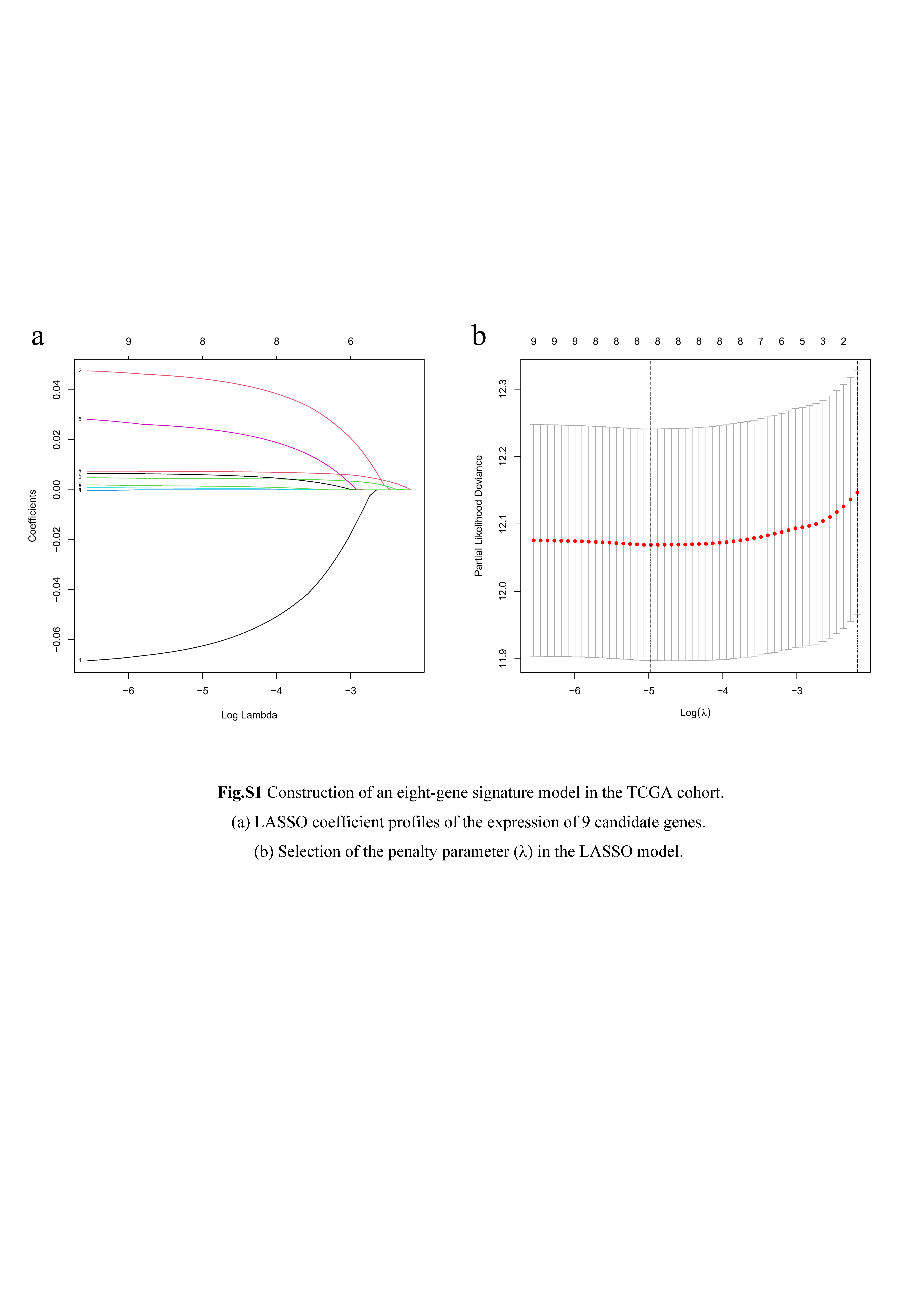

Supplement: Supplementary file 1 — Supplementary Figure S1. [file 41598_2023_41998_MOESM1_ESM.tif]

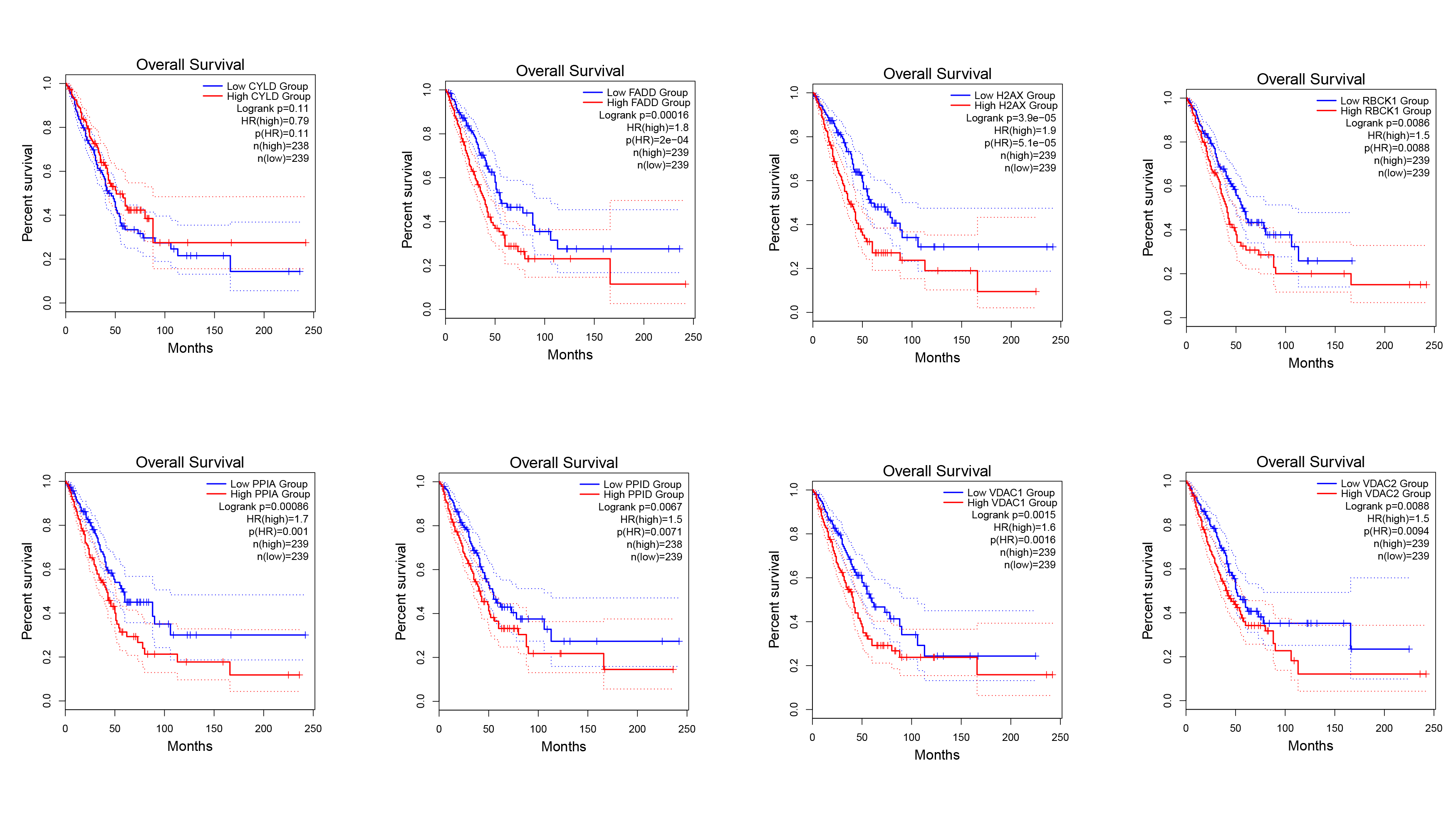

Supplement: Supplementary file 2 — Supplementary Figure S2. [file 41598_2023_41998_MOESM2_ESM.tif]

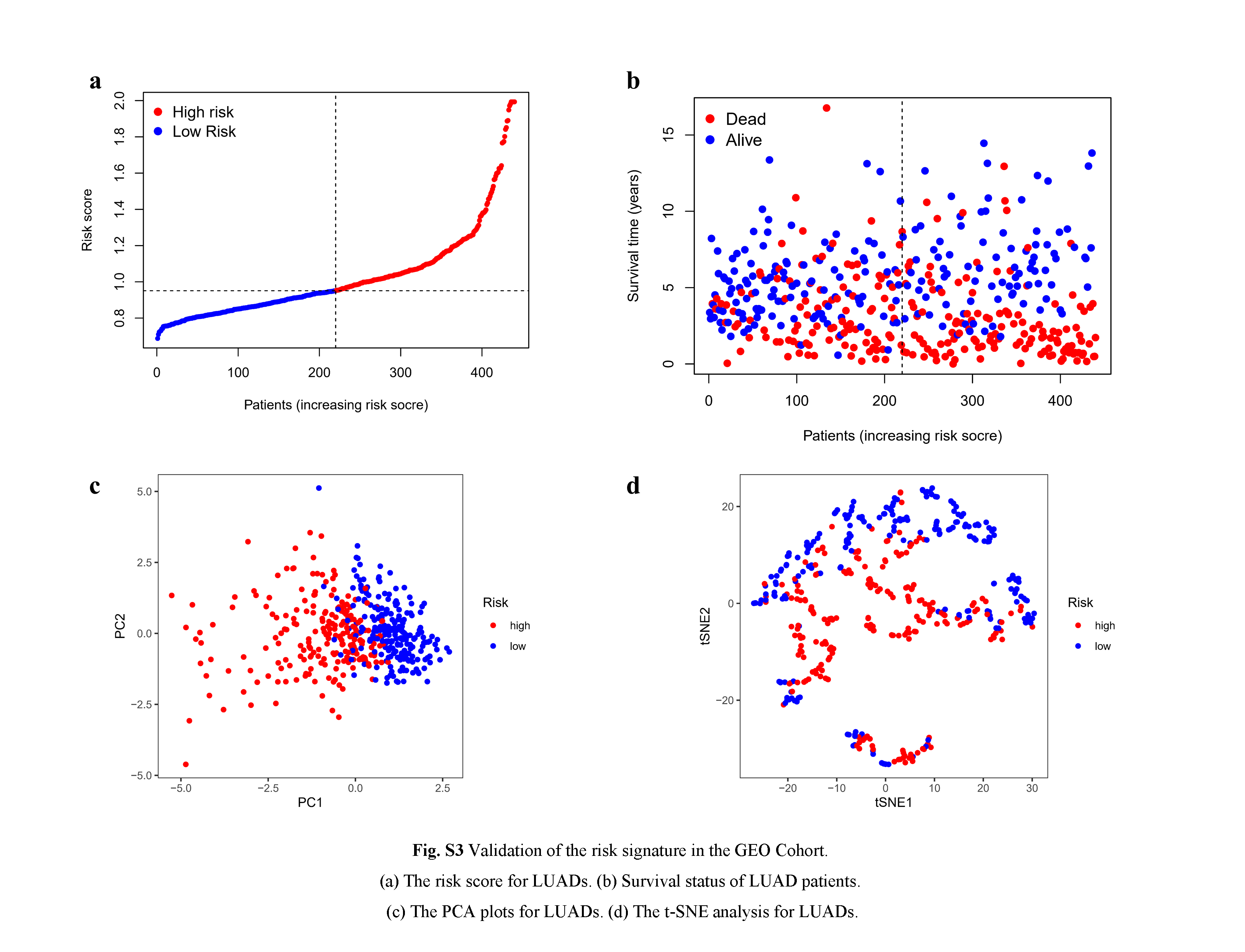

Supplement: Supplementary file 3 — Supplementary Figure S3. [file 41598_2023_41998_MOESM3_ESM.tif]

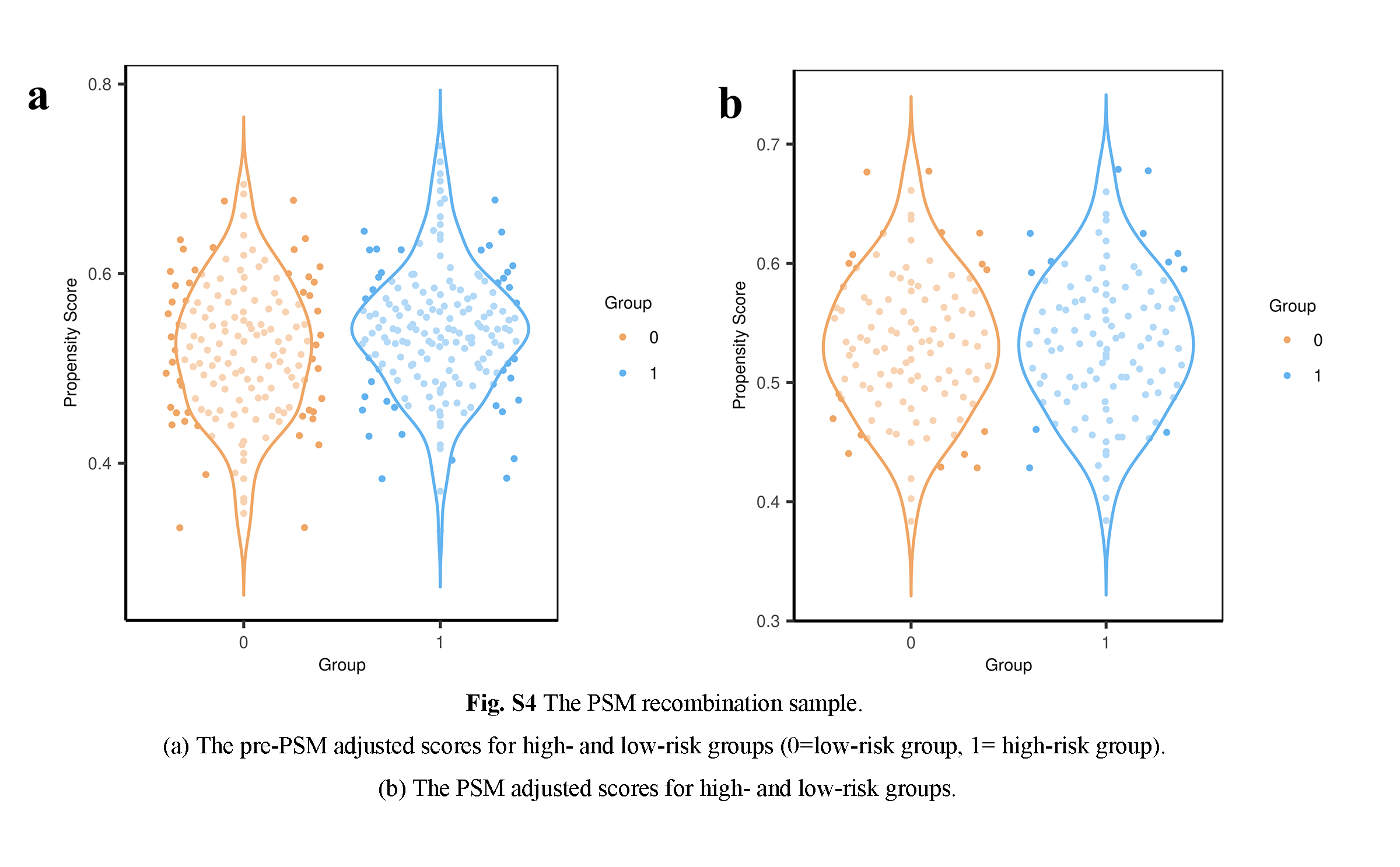

Supplement: Supplementary file 4 — Supplementary Figure S4. [file 41598_2023_41998_MOESM4_ESM.tif]

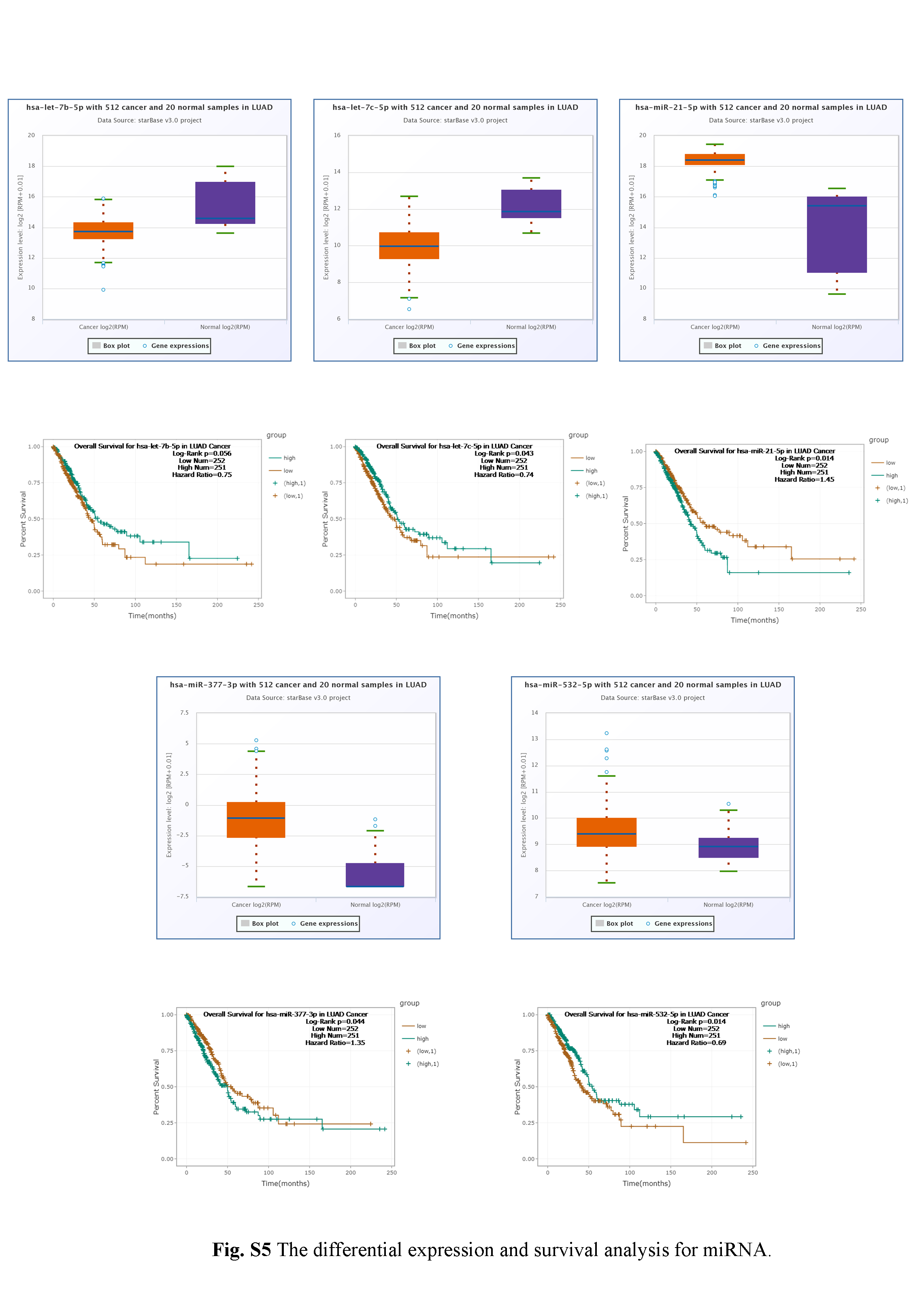

Supplement: Supplementary file 5 — Supplementary Figure S5. [file 41598_2023_41998_MOESM5_ESM.tif]

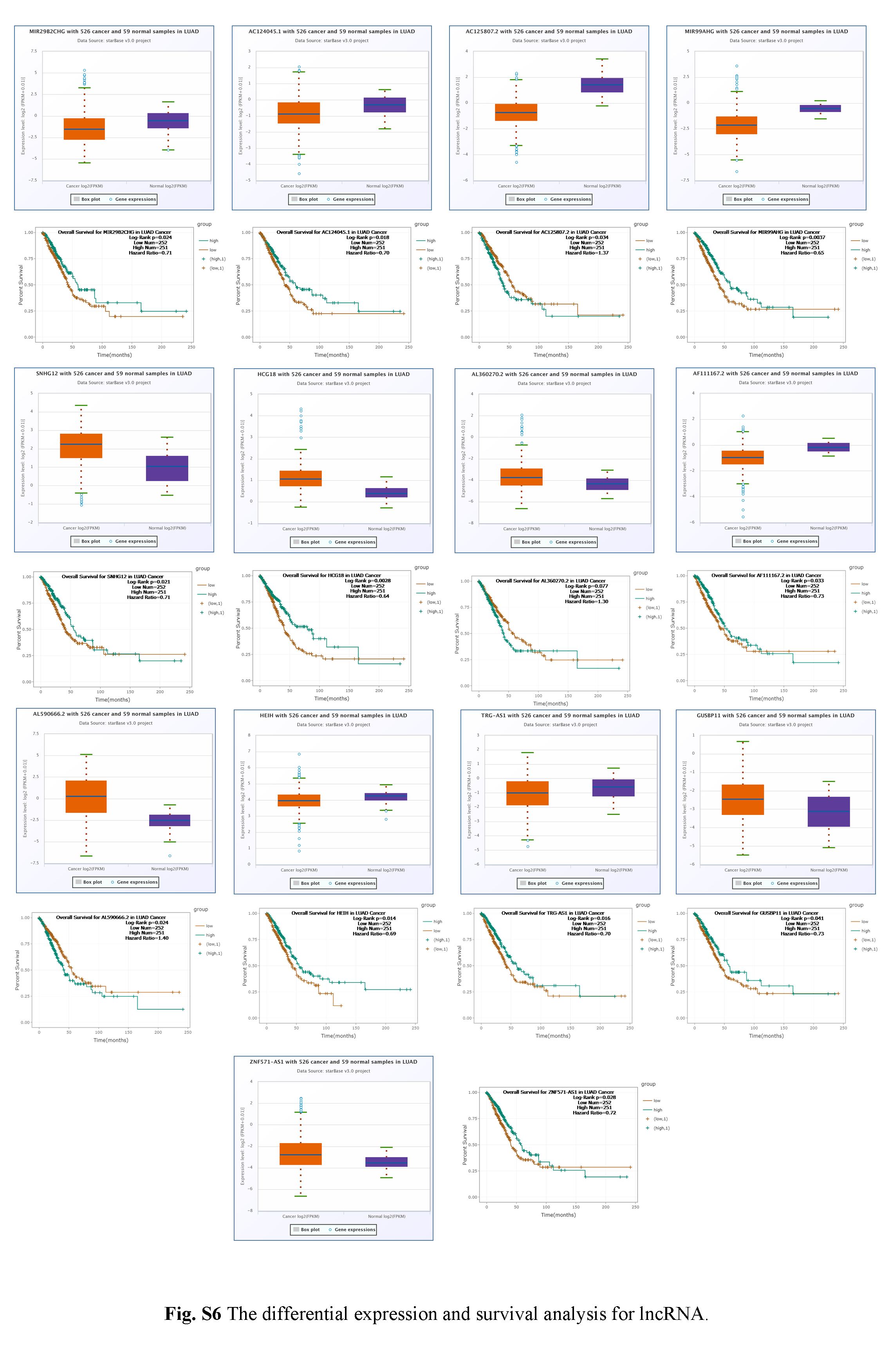

Supplement: Supplementary file 6 — Supplementary Figure S6. [file 41598_2023_41998_MOESM6_ESM.tif]

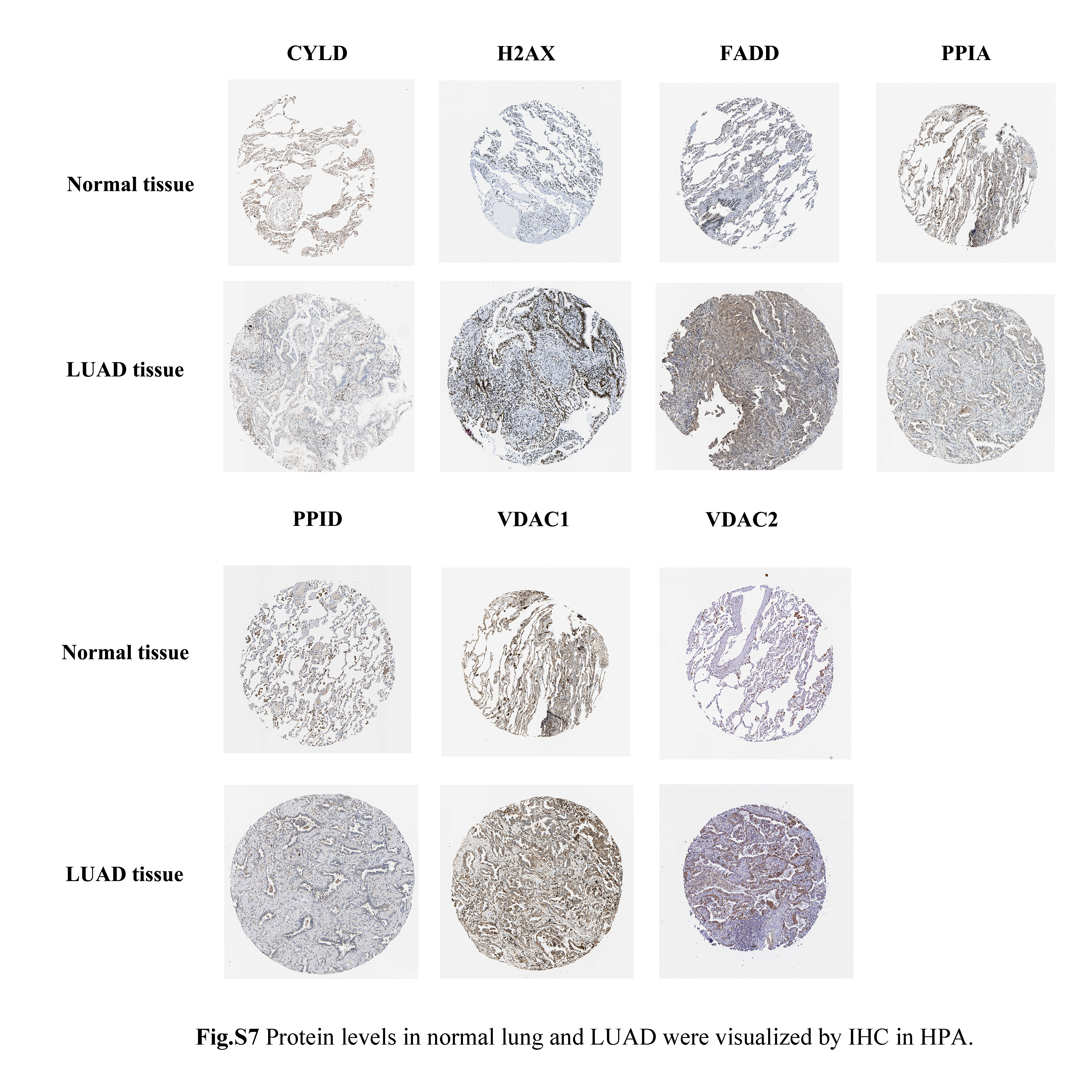

Supplement: Supplementary file 7 — Supplementary Figure S7. [file 41598_2023_41998_MOESM7_ESM.tif]
