# Supplementary material for: A novel necroptosis related gene signature and regulatory network for overall survival prediction in lung adenocarcinoma
Source: Sci Rep. 2023 Sep 15;13:15345. doi: 10.1038/s41598-023-41998-2 (PMC10504370; doi:10.1038/s41598-023-41998-2)
Supplement: Supplementary file 9 — Supplementary Table S2. [file 41598_2023_41998_MOESM9_ESM.docx]

**Table S2 Clinical information and basic characteristics of LUAD patients in GEO cohort after PSM**

| **Variables** | **RISK: low** | **RISK: high** | **SMD** | **P value** |
| --- | --- | --- | --- | --- |
| **Age** | 64.51 ± 9.41 | 63.27± 9.22 |  | 0.326 |
| **Gender** |  |  | 0.073 | 0.686 |
| Female | 54 (49.1) | 58 (52.7) |  |  |
| Male | 56 (50.9) | 52 (47.3) |  |  |
| **Smoke** |  |  | 0.056 | 0.835 |
| No | 12 (10.9) | 14 (12.7) |  |  |
| Yes | 98 (89.1) | 96 (87.3) |  |  |
| **Treatment** |  |  |  | 0.833 |
| No Treatment | 72 (65.5) | 77 (70) | 0.097 |  |
| Adjuvant Chemotherapy | 17 (15.5) | 14 (12.7) | 0.078 |  |
| Adjuvant Radiotherapy | 6 (5.5) | 7 (6.4) | 0.039 |  |
| Adjuvant Radio-chemotherapy | 15 (13.6) | 12 (10.9) | 0.083 |  |
| **T** |  |  |  | 0.755 |
| 1 | 33 (30) | 31 (28.2) | 0.04 |  |
| 2 | 74 (67.3) | 74 (67.3) | 0 |  |
| 3 | 3 (2.7) | 5 (4.5) | 0.097 |  |
| **N** |  |  |  | 0.672 |
| 0 | 71 (64.5) | 77 (70) | 0.116 |  |
| 1 | 25 (22.7) | 22 (20) | 0.067 |  |
| 2 | 14 (12.7) | 11 (10) | 0.086 |  |
